# AI for Health Care Quality and Patient Safety: Scoping Review of Diagnostic, Predictive, and Decision Support Applications

**DOI:** 10.2196/95157

**Published:** 2026-09-21

**Authors:** Yang Xu, Jeremy Veillard, Jude Dzevela Kong

**Affiliations:** 1 Artificial Intelligence and Mathematical Modelling Lab, Dalla Lana School of Public Health, University of Toronto, Room 500, 155 College Street, Toronto, ON, M5T 3M7, Canada, 1 416-978-0901; 2 Institute of Health Policy, Management and Evaluation, University of Toronto, Toronto, ON, Canada; 3 Global South Artificial Intelligence for Pandemic and Epidemic Preparedness and Response Network, Toronto, ON, Canada; 4 Africa-Canada Artificial Intelligence and Data Innovation Consortium, Toronto, ON, Canada; 5 Munk School of Global Affairs and Public Policy, University of Toronto, Toronto, ON, Canada; 6 Department of Mathematics, Bahen Centre for Information Technology, University of Toronto, Toronto, ON, Canada

**Keywords:** AI, health care, clinical decision support, diagnostic accuracy, implementation science

## Abstract

**Background:**

AI systems have achieved strong performance on discrete diagnostic, predictive, and decision support tasks; yet, translation into sustained clinical benefit remains uneven. Existing reviews often examine single application areas, obscuring barriers that operate across the health care AI ecosystem.

**Objective:**

This review aimed to map the published evidence on AI for health care quality and patient safety across application domains, characterize evidence maturity, and identify cross-domain barriers that limit translation into clinical value.

**Methods:**

We conducted a scoping review following Joanna Briggs Institute methodology and reported it according to PRISMA-ScR (Preferred Reporting Items for Systematic Reviews and Meta-Analyses extension for Scoping Reviews), with search documentation guided by PRISMA-S (Preferred Reporting Items for Systematic Reviews and Meta-Analyses literature search extension). Five databases (MEDLINE via PubMed, Scopus, Web of Science Core Collection, IEEE Xplore, and CINAHL Plus with Full Text) were searched for English-language records published from January 1, 2017, to April 30, 2026. Eligibility followed the population-concept-context framework. Application domains were coded nonmutually. World Health Organization (WHO) quality of care dimensions served as a deductive scaffold, and recurring constraints were grouped into cross-domain barriers. Methodological characteristics were charted descriptively, informed by validation approach, implementation context, and established AI reporting frameworks.

**Results:**

The search identified 43,394 records, of which 275 were retained in the core charting corpus after staged screening. The evidence covered 4 nonmutually coded domains: diagnostic AI (n=142, 51.6%), predictive analytics (n=192, 69.8%), clinical decision support or implementation-related applications (n=233, 84.7%), and economic or value assessment (n=53, 19.3%). It was broad but uneven in maturity, with the same gaps across all 4 domains. Prospective and external validation were uncommon, and retrospective results often lacked evidence of improved care. Workflow or process measures were recorded in only 13 (4.7%) records, and explicit equity or subgroup analyses were recorded in 20 (7.3%) records, indicating limited measurement of real-world impact. Economic and governance evidence was also thin, with formal economic evaluations found in 4 (1.5%) records and little attention to monitoring models after deployment. These gaps indicate limited evidence on how AI performs in clinical practice.

**Conclusions:**

We identified 5 recurring barriers to translating health care AI into clinical value: limited prospective external validation, workflow-mediated effectiveness, infrastructure fragmentation, equity and generalizability deficits, and economic and governance uncertainty. Unlike domain-specific reviews, this review maps evidence across the 4 domains, locates the barriers within the WHO quality of care dimensions, and distinguishes descriptive findings from interpretive synthesis. The binding constraint on clinical value is no longer primarily technical but structural, recurring across domains. Health systems and regulators should therefore treat prospective external validation, workflow-integrated deployment, infrastructure readiness, equity monitoring, and lifecycle governance as prerequisites for responsible adoption.

## Introduction

### Background

AI has become one of the most actively developed areas of modern medicine. Over the past decade, AI systems based on deep learning have shown strong performance across a range of clinical tasks, from the interpretation of medical images to the prediction of patient deterioration [1,2]. Three developments have driven this growth, namely the rapid expansion of available health care data, advances in machine learning methods, and the computational capacity needed to process complex medical information at scale [3].

The potential of AI in health care extends beyond automation. It has been argued that AI could help address several long-standing problems, such as reducing diagnostic errors [4], mitigating cognitive bias in clinical decisions, extending specialist expertise to underserved populations, and improving outcomes while containing costs [5-7]. Interest intensified during the COVID-19 pandemic, when predictive models were deployed rapidly in resource-constrained settings [8]. This enthusiasm, however, is increasingly set against the difficulty of moving AI from development into routine care. Although thousands of studies describe AI algorithms for clinical use, only a small fraction have advanced to prospective validation or real-world deployment [9]. The persistent distance between a working algorithm and demonstrated clinical utility, sometimes described as an “AI chasm,” is not explained by any single shortcoming. It reflects a set of recurring obstacles that span how AI is validated, how it fits into clinical work, and how the systems and institutions around it are resourced and governed.

At the center of this situation is a paradox. Individual studies routinely report strong performance and favorable economic projections, yet the evidence base as a whole remains immature where it matters most for clinical use. Many studies rely on retrospective designs with a high risk of bias while reporting accuracy that rivals or exceeds that of human experts [10]. Prospective external validation, which is the test most relevant to real-world reliability, has been carried out for only a small share of published systems [11]. Economic evaluations frequently project savings but omit implementation costs and depend on static assumptions that may overstate benefit [12]. Questions of equity remain largely unresolved, with little evidence on how AI affects different patient populations and whether it narrows or widens existing disparities [13,14]. Taken together, these gaps suggest that the limits on clinical value lie less in algorithmic capability than in how AI is evaluated, integrated, resourced, and governed.

Understanding this paradox is critical for several stakeholders. Health care administrators must make informed decisions about substantial AI investments with uncertain returns. Clinicians require evidence to determine how AI tools will integrate into existing workflows and affect patient care. Policymakers need frameworks to regulate AI applications appropriately, and patients deserve transparent information about the benefits and risks of AI-assisted care.

Recent reviews have clarified important parts of this evidence base, but most remain organized around a single application area or evidence type. For example, existing reviews have examined diagnostic AI in imaging and digital pathology [15], a broad scoping review of AI for patient safety [16], machine learning models for sepsis prediction [11], AI-supported optimization of medication alerts in clinical decision support systems [17], and the methodology and reporting of economic evaluations of health care AI [18,19]. These reviews show that the translation problem is not confined to one specialty or model class. However, because they focus on specific domains, they are less able to identify barriers that recur across diagnostic, predictive, decision support, and value assessment applications. A cross-domain scoping review is therefore needed to map the breadth of evidence, compare maturity across application areas, and identify shared constraints on translation into quality and patient safety value.

This scoping review addressed that gap by mapping the evidence on AI for health care quality and patient safety across 4 application domains, namely diagnostic AI, predictive analytics, clinical decision support, and economic or value assessment. A deliberately broad scope allowed constraints that cut across domains, in validation, workflow integration, infrastructure, equity, and governance, to be characterized as shared features of the field rather than as isolated problems within one specialty. A scoping review is well suited to this aim because the objective is to map evidence and identify patterns rather than to estimate effects and because a narrower design would fragment the question across several separate analyses. To organize quality- and safety-relevant findings, we used the World Health Organization (WHO) quality of care dimensions as a guiding framework [20,21]. The review had 3 objectives. The first is to map the available evidence on AI across the 4 application domains and characterize its maturity. The second is to identify the recurring barriers that limit translation into clinical practice. The third is to synthesize these barriers into a coherent account that can inform research priorities, implementation, and governance for trustworthy health care AI.

### Review Question

This review addressed 2 questions. First, what is the scope, distribution, and evidence maturity of published research on AI across the diagnostic, predictive analytics, clinical decision support, and economic or value assessment domains in health care? Second, what recurring barriers limit the translation of this evidence into clinical quality and patient safety value across these domains?

## Methods

### Protocol, Registration, and Reporting

This scoping review was conducted in accordance with the Joanna Briggs Institute methodology for scoping reviews [22] and is reported following the PRISMA-ScR (Preferred Reporting Items for Systematic Reviews and Meta-Analyses extension for Scoping Reviews) [23-25]. Search reporting was guided by PRISMA-S (Preferred Reporting Items for Systematic Reviews and Meta-Analyses literature search extension) [26] to improve transparency and reproducibility of the literature search. No protocol was formally registered. The absence of protocol registration is reported as a limitation; however, the eligibility criteria, search documentation, staged screening workflow, and charting procedures are reported in the following sections and in the appendices to support auditability and replication. The PRISMA-ScR checklist is provided as Checklist 1.

### Eligibility Criteria

Eligibility was structured using the population-concept-context framework. The population included patients, clinicians, and other actors in any health care delivery setting, including hospital, community, and public health contexts. The concept was AI or machine learning relevant to health care quality or patient safety, spanning diagnostic AI, predictive analytics or early warning, clinical decision support, implementation, validation, equity, and economic or value assessment. The context was any real-world or simulated clinical environment, including external validation cohorts and retrospective or prospective evaluations. Eligible records were English-language, peer-reviewed publications from January 1, 2017, to April 30, 2026. Full inclusion and exclusion criteria, organized by population, concept, and context, are detailed in Table 1. For this review, real-world health care settings were defined as clinical or health system environments in which AI systems were evaluated using patient care data, clinical workflows, health system records, external validation cohorts, or deployment contexts that reflected actual care delivery. Simulated clinical environments were defined as controlled test settings, reader studies, retrospective workflow simulations, or scenario-based evaluations designed to assess clinical feasibility, diagnostic or decision support performance, or workflow relevance without routine care deployment. Simulated studies were eligible only when they used clinical data or clinically realistic tasks and addressed health care quality, patient safety, diagnosis, prediction, decision support, implementation, or economic value.

**Table 1.** Inclusion and exclusion criteria for the scoping review, structured using the population-concept-context framework^a^.

Study characteristics	Inclusion criteria	Exclusion criteria
Language	English-language records.	Non-English records.
Period	Published between January 1, 2017, and April 30, 2026.	Published before January 1, 2017, or after April 30, 2026.
Publication type	Full-length peer-reviewed journal articles, review articles with sufficient evidence synthesis content, systematic reviews, scoping reviews, meta-analyses, economic evaluations, validation studies, implementation studies, and full-length conference papers or proceedings papers with sufficient methodological and results information for eligibility assessment and data charting.	Editorials, commentaries, opinion pieces, viewpoints, letters, news items, magazine articles, books, book chapters, standards, courses, protocols without results, conference abstracts, posters, meeting summaries, or short proceedings abstracts without sufficient methods and results.
Population and care context	Studies involving human health care settings, including hospital, clinical, community, nursing, allied health, public health, and health system contexts.	Animal-only studies, laboratory-only studies, nonhuman studies, or studies outside health care.
Concept: AI technology	Studies evaluating or reviewing AI, ML^b^, deep learning, neural networks, large language models, foundation models, computer-aided diagnosis or detection, clinical prediction models using AI or ML, or AI-enabled clinical decision support.	Studies not involving AI or ML; studies using only conventional statistical modeling without an AI or ML component, such as standard logistic regression, Cox regression, linear regression, rule-based scores, or traditional risk scores, unless explicitly embedded within or compared against an AI or ML approach; and studies where AI was mentioned only as background but not evaluated or synthesized.
Concept: quality and safety relevance	Studies had to address at least one health care quality, patient safety, diagnostic, predictive, clinical decision support, implementation, workflow, validation, equity, or economic value dimension. Eligible examples included predictive accuracy, external or prospective validation, alert performance, medication safety, medical errors, adverse drug events, workflow integration, clinical implementation, cost-effectiveness, return on investment, or quality of care outcomes.	Studies with no clear relevance to health care quality, patient safety, diagnosis, prediction, clinical decision support, implementation, workflow, validation, equity, or economic value outcomes.
Application domain	Studies related to 1 or more of the review’s 4 target AI application domains: diagnostic AI, predictive analytics, including early warning models, clinical decision support systems, or economic evaluation or value assessment of AI in health care.	AI studies outside the review scope, including purely technical model development, image processing, segmentation, feature extraction, engineering optimization, educational AI, administrative automation, or operational analytics without direct clinical quality, safety, diagnostic, predictive, decision support, implementation, or economic relevance.
Setting	Real-world health care settings, clinical datasets, external validation cohorts, prospective or retrospective clinical evaluations, implementation settings, or simulated clinical environments designed to evaluate clinical feasibility, workflow, safety, or decision support relevance.	Purely theoretical, conceptual, or technical settings without clinical data, patient care relevance, implementation relevance, or health system relevance.
Evidence requirements	Records had to provide sufficient information on the AI application, study context, methods, and outcomes to support data charting. Reviews and meta-analyses were eligible as secondary evidence but were distinguished from primary empirical studies during data extraction.	Records lacking sufficient accessible methodological or outcome detail to support eligibility assessment or data charting; retracted publications; errata or corrections without original study data.

a Criteria were applied to English-language records published from January 1, 2017, to April 30, 2026, addressing AI or ML relevant to health care quality or patient safety.

b ML: machine learning.

### Information Sources and Search Strategy

Searches were conducted in 5 bibliographic databases, including MEDLINE via PubMed, Scopus, Web of Science Core Collection, IEEE Xplore, and CINAHL Plus with Full Text. These databases were selected to cover biomedical, multidisciplinary, engineering, nursing, allied health, and health technology literature relevant to AI applications in health care quality and patient safety. The search covered records published from January 1, 2017, to April 30, 2026, and was limited to English-language records.

Search strategies combined controlled vocabulary and free-text terms across 2 concept blocks. The first block captured AI and machine learning techniques, with terms such as artificial intelligence, machine learning, deep learning, neural networks, large language models, and foundation models. The second block captured health care quality, patient safety, and related clinical applications, with terms such as patient safety, quality of care, health-care quality, clinical decision support, decision support systems, medical errors, and medication errors. Controlled vocabulary was applied where available, including MeSH terms in MEDLINE via PubMed. Search syntax was adapted for each database. Complete database-specific search strings, dates of execution, applied limits, and export notes are provided in Multimedia Appendix 1 in accordance with the PRISMA-S guideline [26]. Publication-type eligibility was enforced primarily during screening. As publication-type indexing is not equivalent across platforms, MEDLINE via PubMed additionally used controlled publication-type tags to exclude editorials, comments, letters, and news records at the query stage, whereas for Scopus, Web of Science, IEEE Xplore, and CINAHL, these exclusions were applied uniformly during the staged screening workflow.

Database hit counts were used for PRISMA (Preferred Reporting Items for Systematic Reviews and Meta-Analyses) identification counts. The final database-level counts were MEDLINE via PubMed (n=16,561, 38.2%), Scopus (n=15,145, 34.9%), Web of Science Core Collection (n=7163, 16.5%), IEEE Xplore (n=3141, 7.2%), and CINAHL Plus with Full Text (n=1384, 3.2%), for a total of 43,394 records identified before deduplication.

### Record Management and Deduplication

Records from each database were exported and combined for local reference management. Among the 43,394 records identified, 43,357 (99.9%) were parsed and imported for deduplication; 37 (0.1%) records were not imported owing to a documented Scopus export and import discrepancy and are recorded in the audit log rather than substituted for database hit counts. Deduplication was then performed locally using a conservative rule-based workflow, which removed 15,692 (36.2%) duplicate records and left 27,665 (63.8%) unique records for staged screening. The deduplication rules and the database-level export, import, and deduplication counts are documented in Multimedia Appendix 1.

### Selection of Sources of Evidence

As the search produced a large record set, study selection followed a staged process combining rule-based filters with manual review. Stage A comprised 2 deterministic substeps, described as stage A0 and stage A1 in Multimedia Appendix 2. Stage A0 applied high-confidence structural rules based on the eligibility criteria in Table 1, including rules for retracted or correction records, excluded publication types, protocols without reported results, short conference abstracts, obvious non–health care contexts, and purely technical studies without relevant clinical or quality and safety signals. This structural prescreen excluded 2725 (9.8%) of 27,665 records and retained 24,940 (90.2%) records for further stage A processing. Stage A1 then applied additional deterministic exclusion rules, excluding a further 7521 (30.2%) records and giving a cumulative total of 10,246 (37%) rule-based exclusions. Scope signal prioritization was then applied to the remaining 17,419 (69.8%) records.

For stage A scope signal prioritization, the title and available abstract of each of these 17,419 records were assessed against 5 groups of signals: patient safety and quality, clinical decision support, diagnostic AI and clinical evaluation, predictive analytics, and implementation, economic, or equity content. Each group contributed a maximum of 1 point, giving a possible score of 0 to 5. Thresholds of at least 1, 2, or 3 matched groups were evaluated. The archived selection rule targeted a manual screening pool of 1000 to 3000 records. As the threshold of at least two groups produced 5564 candidates, exceeding the upper limit of 3000, the threshold was set at 3 or more matched groups. This produced a high-signal pool of 1378 records for manual title and abstract screening. The remaining 16,041 (92.1%) records matched fewer than 3 groups and were retained in the audit trail but were not advanced to manual screening. Among the 1378 (7.9%) manually screened records, 96 (7%) were excluded, 1246 (90.4%) were classified as eligible, and 36 (2.6%) were classified as uncertain. Records classified as eligible or uncertain were retained for stage B to avoid premature exclusion when eligibility could not be determined confidently from the title and abstract alone, resulting in 1282 retained records.

The 1282 retained records then underwent stage B evidence feature prioritization using deterministic matching of archived trigger strings. A record was assigned to tier A and advanced when it contained at least 1 archived tier A trigger related to prospective or randomized evaluation, review-level synthesis, implementation or deployment, or economic or value assessment. When no tier A trigger was present, records containing at least one tier B trigger related to external or broader validation, multicenter or multisite scale, registry or population-scale evidence, or clinician or reader comparison were assigned to tier B. Records with neither a tier A nor tier B trigger were assigned to tier C. The 377 (29.4%) tier A records were carried forward. The remaining 905 records, comprising tier B, tier C, and 1 record without a verifiable archived tier assignment, were retained in the audit trail but were not advanced.

The 377 tier A records then entered a final title- and abstract-based charting prioritization. Each record received a weighted score combining signals for direct patient safety or quality relevance, clinical decision support, prospective or deployment evaluation, external or multicenter validation, secondary evidence synthesis, and predictive or diagnostic evaluation, with penalties applied where the evidence signal appeared only as a future need or limitation, where the record was a broad nonspecific review, or where the content was primarily educational, perception based, or contextual. Records reaching a score of at least nine or carrying an explicit economic or value assessment signal were advanced to detailed assessment and charting. This rule advanced 276 (73.2%) records. A further 99 (26.3%) records scored below the threshold without an explicit economic or value assessment signal and were retained in the audit trail without being advanced, and 2 (0.5%) records were excluded at this stage, 1 because it had no eligible AI or machine learning focus and 1 because it concerned a nonhuman or veterinary health care context. The prioritization score allocated charting effort and was not an appraisal of study quality, risk of bias, or certainty of evidence. Multimedia Appendix 2 provides the detailed rules for each prioritization stage, including the signals, thresholds, and scoring weights used, together with record-level dispositions for the 1282 records entering stage B and the 377 records entering the final charting prioritization.

Among the 276 reports advanced to detailed assessment and charting, 1 duplicate report was identified during final reconciliation and removed, leaving 275 reports in the core charting corpus. Eligibility was enforced upstream through the deterministic rules and the manual title and abstract screening described earlier, and the 2 eligibility-based exclusions were applied at the final title- and abstract-based charting prioritization. All 276 reports that entered report-level assessment therefore already met the eligibility criteria, and the only report removed at that stage was the duplicate identified during final reconciliation. All inclusion decisions at the manual screening and detailed assessment stages were made by the review team based on the eligibility criteria.

The deterministic stage A and stage B screening and prioritization rules were applied programmatically. Manual screening, full-text assessment, and data charting were conducted primarily by 1 reviewer (YX), with periodic verification by 2 additional reviewers (JV and JDK) who independently checked subsets of records to confirm consistency with the eligibility criteria and charting fields. These checks focused on eligibility consistency, domain assignment, and selected charting fields rather than serving as a formal independent dual-reviewer calibration exercise. The verification subsets were drawn across the staged review process rather than through a predefined sampling scheme, and formal concordance rates or κ statistics were not calculated. The interpretation and synthesis of the charted evidence were reviewed and discussed by the full author team. To reduce error and support reproducibility, the screening process used predefined eligibility criteria, deterministic rule-based filters, retained audit logs, and structured charting fields. The reliance on a single primary reviewer is reported as a limitation.

### Data Charting Process and Data Items

A standardized charting form was developed for the 275 records in the core charting corpus. Charted variables included bibliographic details, study design or evidence type, validation maturity, deployment or real-world context, workflow or process metrics, equity or subgroup indicators, infrastructure indicators, governance or lifecycle indicators, and economic or value assessment characteristics where applicable. The full corpus, including bibliographic details, persistent source links, application domain labels, abstracts, row-level evidence maturity fields, barrier-related indicators, economic evaluation fields, and audit checks for figure and count reproducibility, is provided in Multimedia Appendix 3. The counts and classifications presented in the evidence visualizations were derived directly from these row-level charting fields and the associated count check procedures in Multimedia Appendix 3, rather than from separate manual counts.

Each record was coded for 1 or more of 4 application domains using prespecified operational definitions, with nonmutually exclusive coding to reflect that many records addressed more than one AI function or care delivery context. Diagnostic AI was defined as the classification, detection, or characterization of a clinical condition from imaging, pathology, or physiological signal data, with diagnostic performance as the primary outcome [9,15]. Predictive analytics was defined as the forecasting of a future clinical event or patient trajectory from longitudinal or time series data, with predictive performance or downstream clinical impact as the primary outcome [11,27,28]. Clinical decision support or implementation-related applications were defined as AI generating actionable recommendations, alerts, or guidance to clinicians at the point of care, or studies of how such systems are implemented in practice [17,29]. Economic or value assessment was defined as studies whose primary contribution was a cost, cost-effectiveness, budget impact, return on investment, or value analysis of an AI intervention [18,19]. Domain counts therefore sum to more than 275 across the 4 categories and should not be interpreted as mutually exclusive classifications.

For selected cross-domain indicators, rapid title, abstract, and metadata charting was supplemented by targeted manual verification of flagged records. These indicators included validation maturity, workflow or process metrics, infrastructure constraints, equity or subgroup signals, governance or lifecycle content, and economic evidence type. Indicator counts therefore reflect the visibility of selected features in the published record rather than formal adjudication of study-level conduct and may undercount features reported only in full texts.

### Methodological Characterization

Consistent with scoping review methodology, no formal risk-of-bias appraisal was used to exclude records. Instead, methodological characteristics were charted descriptively to support the evidence map and the cross-domain synthesis. These characteristics included study design or evidence type, validation approach with a graded record of internal, external, multicenter, prospective, and randomized evaluation, deployment or real-world context, workflow or process metrics, and economic evaluation type. Established frameworks for AI prediction model reporting and risk-of-bias assessment, including the TRIPOD+AI (Transparent Reporting of a Multivariable Prediction Model for Individual Prognosis or Diagnosis extension for Artificial Intelligence) [27] and the PROBAST (Prediction model Risk of Bias Assessment Tool) [28], were used only as descriptive lenses to inform interpretation of reporting and validation issues. They were not applied as formal scoring or exclusion criteria.

### Synthesis of Results

Synthesis proceeded in 2 stages. First, the charted evidence was summarized descriptively across the 4 application domains of diagnostic AI, predictive analytics or early warning, clinical decision support or implementation-related applications, and economic or value assessment. The WHO quality of care dimensions were used as a deductive organizing scaffold for quality- and safety-relevant outcomes [20,21].

Second, recurring cross-domain barriers were developed through inductive synthesis of the charted evidence, drawing on the principles of framework synthesis and thematic synthesis [30,31]. Candidate themes were compared across domains and consolidated into 5 barriers, namely the validation gap, workflow-mediated effectiveness, infrastructure fragmentation, equity and generalizability deficits, and economic and governance uncertainty. These barriers are presented as interpretive synthesis themes rather than formal per-record labels. Implementation science concepts from the NASSS (nonadoption, abandonment, scale-up, spread, and sustainability) framework, the Consolidated Framework for Implementation Research, the RE-AIM (reach, effectiveness, adoption, implementation, and maintenance) framework, and sociotechnical models were used as interpretive anchors when discussing workflow integration, adoption, implementation fidelity, and sustainability [32-34]. These frameworks were not applied as separate coding frameworks or formal appraisal tools. Supporting background references used to define methodological approaches, reporting frameworks, health care quality concepts, and implementation science constructs were kept distinct from the formal 5-database scoping review corpus and were not counted in the PRISMA flow.

## Results

### Study Selection and Overview

**Figure 1.**
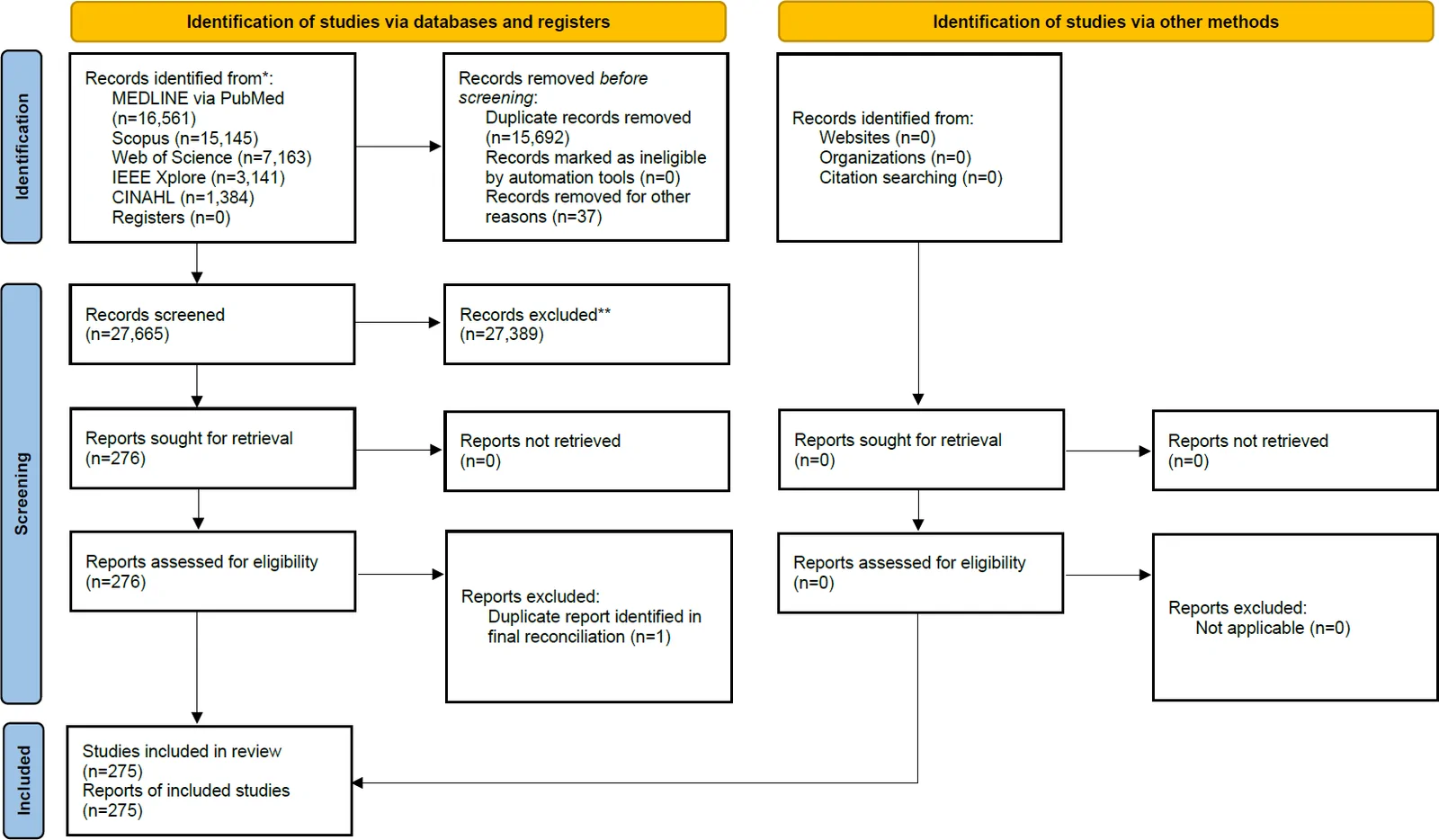
PRISMA (Preferred Reporting Items for Systematic Reviews and Meta-Analyses) 2020 flow diagram of study selection. Records were identified from 5 bibliographic databases (MEDLINE via PubMed, Scopus, Web of Science Core Collection, IEEE Xplore, and CINAHL Plus with Full Text) for English-language publications from January 1, 2017, to April 30, 2026. No registers or other sources were searched; the corresponding entries are reported as zero. Records not advanced at any stage were retained in the audit trail rather than discarded. Before report-level assessment, 27,389 records were excluded or not advanced: 10,246 by deterministic rule-based criteria, 16,041 after stage A scope signal prioritization, 96 during manual title and abstract screening, 905 after stage B evidence feature prioritization, and 101 during the final title- and abstract-based charting prioritization, of which 99 did not reach the archived selection threshold, and 2 were excluded on eligibility grounds. *Numbers are reported separately for each database and register rather than as a combined total. **No records were excluded solely by automation tools; all exclusions were manually verified. Source: Page MJ, et al. BMJ. 2021;372:n71. doi:10.1136/bmj.n71. The PRISMA 2020 flow diagram template is licensed under CC BY 4.0 [35]. The staged screening and prioritization procedure is described in the *Methods* section and in Multimedia Appendix 2.

Searches of 5 bibliographic databases identified 43,394 records: MEDLINE via PubMed (n=16,561, 38.2%), Scopus (n=15,145, 34.9%), Web of Science Core Collection (n=7163, 16.5%), IEEE Xplore (n=3141, 7.2%), and CINAHL Plus with Full Text (n=1384, 3.2%). After reconciliation of an export and import discrepancy involving 37 records and removal of duplicate records (n=15,692, 36.2%), 27,665 records remained for staged screening. A deterministic rule-based workflow excluded 10,246 (37%) records, and 16,041 (92.1%) of the remaining 17,419 records were not advanced after the archived stage A scope signal prioritization threshold. The remaining 1378 (7.9%) high-signal records were manually screened at the title and abstract level, of which 96 (7%) were excluded, and 1282 (93%) were retained for stage B evidence feature prioritization. From these, 905 (70.6%) records were not advanced and were retained in the audit trail, leaving 377 (29.4%) tier A records for the final charting prioritization. Of these, 276 (73.2%) met the archived rule, defined as a composite selection score of at least nine or an explicit economic or value assessment signal, and proceeded to detailed assessment and charting. Ninety-nine (26.3%) records fell below the threshold and carried no such signal and were not advanced. Two (0.5%) further records were excluded on eligibility grounds at this stage, 1 for having no eligible AI or machine learning focus and 1 for a nonhuman or veterinary health care context. Removal of 1 duplicate during final reconciliation left 275 reports in the core charting corpus. The study selection process is shown in Figure 1 .

As many records addressed more than one AI function or care delivery context, application domains were coded nonmutually. The 275-record core charting corpus included diagnostic AI records (n=142, 51.6%), predictive analytics or early warning records (n=192, 69.8%), clinical decision support or implementation-related records (n=233, 84.7%), and economic or value assessment records (n=53, 19.3%). The complete corpus is provided in Multimedia Appendix 3. These domain counts should not be summed because individual records could contribute to more than one application domain. To aid interpretation of the mapped evidence, Table 2 summarizes the distribution and selection rationale for illustrative included records across the 4 application domains. Multimedia Appendix 4 presents the full set of 16 illustrative records, organized separately as 7 (43.8%) primary empirical or economic records and 9 (56.3%) secondary evidence syntheses. For each record, the appendix reports the domain group, record identifier, study design, sample size or evidence base, DOI or source link, AI application, charted contribution, and relevant evidence feature. These records are not presented as a quality-ranked subset or as a statistically representative sample of the full corpus. The 16 records were purposively selected to cover all 4 application domains and both evidence functions. The division between primary and secondary evidence follows the study design field of the charting matrix in Multimedia Appendix 3, and the ratio of 9 secondary to 7 primary records approximates the composition of the full corpus, in which 169 records were reviews or evidence syntheses, and 106 were primary empirical or economic evaluation records. The set was limited to 16 to keep the supplementary presentation readable; the complete 275-record charting corpus remains available in Multimedia Appendix 3.

**Table 2.** Summary of the distribution and selection rationale for illustrative included records across the 4 application domains in the 275-record core charting corpus^a^.

Domain group	Nonmutually coded records in the corpus (n=275), n (%)	Illustrative records selected (n=16), n (%)	Selection emphasis
Diagnostic AI	142 (51.6)	4 (25)	External validation, diagnostic accuracy synthesis, and deployment readiness
Predictive analytics or early warning	192 (69.8)	4 (25)	Prospective evaluation, multicenter validation, and early warning implementation
Clinical decision support or implementation	233 (84.7)	5 (31.3)	Medication safety, alert burden, EHR^b^-integrated CDSS^c^, and workflow and process indicators
Economic or value assessment	53 (19.3)	3 (18.8)	Economic or value evidence, safety or value synthesis, and prospective or modeled evaluation

a Each illustrative record appears once under a single primary display domain. This display assignment is used only for presentation in this table and does not change the nonmutually exclusive domain counts reported in the text, in which a record may contribute to more than one domain. Illustrative records were selected to show the range of domain coverage and complementary evidence functions, not to rank study quality, and no formal risk-of-bias appraisal was used to select or weight records. The full set of 16 illustrative records is presented in Multimedia Appendix 4.

b EHR: electronic health record.

c CDSS: clinical decision support system.

### Study Characteristics and Evidence Landscape

**Figure 2.**
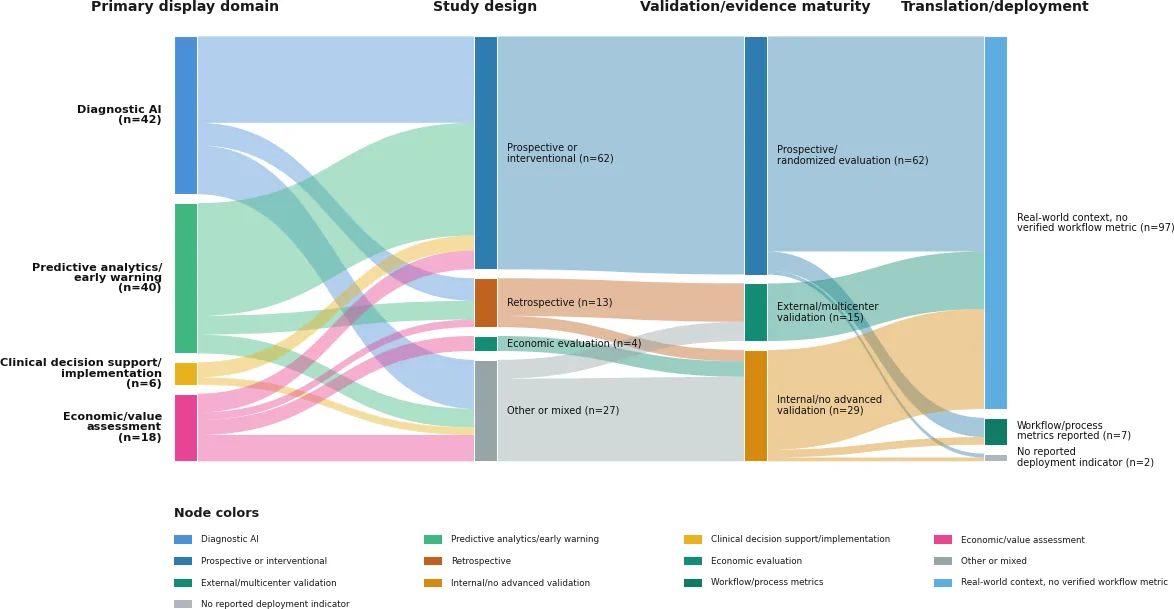
Sankey diagram of evidence characteristics in the empirical and economic evaluation subset of the 275-record core charting corpus (106 records). To improve interpretability of the evidence pathway, review or evidence synthesis records are excluded from this visualization and retained in the full 275-record corpus and in Multimedia Appendix 3. Within the subset, each record is assigned to a single primary display domain, and flows link the primary display domain, study design or evidence type, validation or evidence maturity, and translation or deployment characterization. Study design is shown using 4 broad categories, namely prospective or interventional, retrospective, economic evaluation, and other or mixed. As application domains were otherwise coded nonmutually, the primary display domain is used here for display only and does not replace the nonmutually coded domain counts reported in the text. The “other or mixed designs” category (n=27, 25.5%) denotes included studies whose design was mixed or could not be assigned to a single type. Categories are descriptive charting variables and should not be interpreted as formal study quality ratings or risk-of-bias assessments.

The 275-record core charting corpus was heterogeneous in application domain, evidence type, validation maturity, and translation or deployment characterization. For descriptive visualization, Figure 2 displays the 106 primary empirical and economic evaluation records and excludes review or evidence synthesis records, which improves interpretability of the evidence pathway; review records are retained in the full 275-record corpus and in Multimedia Appendix 3. Within this subset, each record is assigned to a single primary display domain so that it appears once in the Sankey diagram. This display domain variable is used only for visualization and should not be interpreted as replacing the nonmutually coded application domain counts reported earlier. In the display domain classification, the 106 records were assigned to diagnostic AI (n=42, 39.6%), predictive analytics or early warning (n=40, 37.7%), economic or value assessment (n=18, 17%), or clinical decision support or implementation-related applications (n=6, 5.7%).

Evidence type was similarly heterogeneous. Review or evidence synthesis records represented the largest category (169/275, 61.5%), followed by prospective empirical studies (46/275, 16.7%), other or mixed evidence types (27/275, 9.8%), randomized or interventional evaluations (16/275, 5.8%), retrospective empirical studies (13/275, 4.7%), and economic evaluations (4/275, 1.5%). Validation maturity varied across the corpus: 169 (61.5%) review records were categorized as not applicable for primary validation assessment, 62 (22.5%) records reported prospective or randomized evaluation, 15 (5.5%) reported external or multicenter validation, and 29 (10.5%) were categorized as internal validation or no advanced validation reported. Translation and deployment indicators were less frequently documented. Thirteen (4.7%) records reported measurable workflow or process metrics, 249 (90.5%) described a real-world or deployment context without a verified workflow metric, and 13 (4.7%) had no reported deployment indicator. The empirical and economic evaluation subset of these characteristics is visualized in Figure 2.

### Application Domain Evidence Patterns

Across the 275-record core charting corpus, evidence was mapped descriptively rather than pooled quantitatively because study designs, outcome definitions, AI tasks, deployment contexts, and performance metrics were highly heterogeneous. Reported measures included diagnostic accuracy, sensitivity, specificity, area under the receiver operating characteristic curve, alert validity, clinician response, workflow outcomes, cost-effectiveness, return on investment, and broader implementation or governance indicators. These metrics were therefore treated as domain-specific descriptive findings rather than directly comparable estimates of clinical effectiveness.

Diagnostic AI records (142/275, 51.6%) primarily addressed task-specific diagnostic performance in imaging, pathology, endoscopy, radiology, ophthalmology, electrocardiography, and related signal-based applications. This domain contained some of the clearest examples of prospective or randomized evaluation, particularly in image-based screening and procedural decision support, as well as multiple diagnostic accuracy reviews and external validation studies. However, much of the diagnostic AI evidence remained focused on model discrimination or diagnostic test accuracy rather than downstream patient outcomes, workflow effects, equity impacts, or sustained implementation [9,10]. Thus, diagnostic AI provided the most visible examples of advanced evaluation but still illustrated the broader gap between technical performance and clinical value.

Predictive analytics or early warning records (192/275, 69.8%) focused on forecasting clinical deterioration, sepsis, acute kidney injury, cardiac arrest, mortality, heart failure, readmission, and other future patient trajectories. These records frequently reported discrimination or risk stratification metrics and, in selected cases, prospective or interventional evaluation. However, predictive performance was highly dependent on outcome definition, data source, clinical setting, and implementation context. Evidence from this domain therefore supported a recurring pattern: prediction models may perform well in development or validation datasets, but clinical value depends on external validation, site transferability, integration with care team workflows, and whether alerts lead to timely and actionable clinical responses [36].

Clinical decision support or implementation-related records represented the largest nonmutually coded domain (233/275, 84.7%). These records addressed medication safety, alert optimization, diagnostic or therapeutic recommendations, EHR-integrated decision support, patient safety surveillance, workflow support, and implementation challenges. This domain was especially relevant to patient safety because many applications were designed to influence clinician behavior directly. However, measurable workflow or process indicators were uncommon across the corpus: only 13 (4.7%) records reported verified workflow, process, or human-response metrics. Many records described real-world or deployment contexts without reporting concrete metrics such as alert acceptance, override rates, time to action, adherence, documentation burden, or workload. This pattern supports the interpretation that clinical decision support effectiveness is mediated not only by model performance but also by actionability and workflow fit [4].

Economic or value assessment records (53/275, 19.3%) examined cost, cost-effectiveness, return on investment, resource use, efficiency, budget impact, or economic implications of AI adoption. Compared with diagnostic, predictive, and decision support applications, this domain was smaller and more dependent on modeled, retrospective, or secondary evidence. Only 4 (1.5%) of 53 records were formal economic evaluations, and all 4 were conducted in high-income European health systems. Two were cost-effectiveness analyses, one from Spain using a health care–system perspective and lifetime horizon and one from the Netherlands using a societal perspective and lifetime horizon. One was a cost-utility analysis from Germany using a statutory health insurance payer perspective and lifetime horizon, and one was a budget impact analysis from England using a National Health Service perspective and 1-year horizon. These extraction fields are reported at record level in Multimedia Appendix 3. This scarcity of formal economic evaluation is consistent with prior methodological reviews of health care AI economic evaluation [18,19]. Economic findings were therefore interpreted cautiously: reported value claims were treated as descriptive evidence of potential economic impact rather than definitive evidence of realized savings, affordability, or system-level efficiency across health systems.

Taken together, the domain-level findings show that the health care AI literature is broad but unevenly mature. Diagnostic AI and predictive analytics provided the most visible examples of advanced validation and prospective evaluation, whereas clinical decision support and economic or value applications more directly exposed the difficulty of translating AI outputs into workflow-integrated, measurable, and economically credible clinical value. These domain-specific patterns informed the cross-domain synthesis of translation barriers described in the following section.

### Cross-Domain Translation Barriers and Evidence Gaps

#### Overview

Across the core charting corpus, we identified 5 cross-domain barriers that help explain why technical performance does not reliably translate into health care quality or patient safety value. These barriers are the validation gap, workflow-mediated effectiveness, infrastructure fragmentation, equity and generalizability deficits, and economic and governance uncertainty. They were generated through inductive synthesis of the charted evidence and organized using the WHO quality of care dimensions as a deductive scaffold. Table 3 defines each barrier, summarizes the evidence signals charted in this review, and maps each barrier to the implementation science constructs and quality of care dimensions it most directly engages.

**Table 3.** The 5 cross-domain barriers to clinical translation of health care AI identified through inductive synthesis of the 275-record core charting corpus, with their operational definitions, the evidence signals charted in this review, the related implementation science constructs used as interpretive anchors, and the WHO^a^ quality of care dimensions most directly implicated^b^.

Barrier	Operational definition	Evidence signals charted in this review	Related implementation science constructs	WHO quality of care dimensions most directly implicated
The validation gap	Limited prospective, external, or multicenter evaluation before clinical use	Internal validation, retrospective or single-site testing, and limited prospective deployment	NASSS^c^: technology, value proposition, adaptation over time; RE-AIM^d^: effectiveness and maintenance	Effectiveness and safety
Workflow-mediated effectiveness	Clinical benefit depends on whether model outputs connect to executable clinical actions	Alert acceptance, override rates, time to action, clinician response, and workflow fit	NASSS: adopter system and organization; CFIR^e^: inner setting, individuals, and process; RE-AIM: adoption and implementation	Safety, timeliness, people-centeredness, and integration
Infrastructure fragmentation	Fragmented data systems, EHR^f^ architectures, and interoperability limit scalable deployment	Data silos, interoperability gaps, local validation barriers, and model transfer problems	CFIR: inner and outer setting; NASSS: organization and wider context; sociotechnical alignment	Integration, efficiency, and equity
Equity and generalizability deficits	Limited assessment of subgroup performance and transferability across populations	Subgroup reporting, fairness analysis, LMIC^g^ representation, and external population validation	RE-AIM: reach; CFIR: outer setting; NASSS: condition, adopter, wider context	Equity, safety, and effectiveness
Economic and governance uncertainty	Value claims and governance mechanisms remain underdeveloped relative to technical performance	Modeled ROI^h^, missing implementation costs, regulatory uncertainty, lifecycle monitoring, and model drift	NASSS: value proposition, wider context, and adaptation over time; RE-AIM: maintenance; CFIR: outer setting and process	Efficiency and integration

a WHO: World Health Organization.

b The NASSS framework, CFIR, RE-AIM, and sociotechnical concepts are used only as interpretive anchors and were not applied as separate coding frameworks or formal appraisal tools.

c NASSS: nonadoption, abandonment, scale-up, spread, and sustainability.

d RE-AIM: reach, effectiveness, adoption, implementation, and maintenance.

e CFIR: Consolidated Framework for Implementation Research.

f EHR: electronic health record.

g LMIC: low- and middle-income country.

h ROI: return on investment.

#### The Validation Gap

Across diagnostic AI, predictive analytics, clinical decision support, and economic evaluation, high discrimination metrics derived from retrospective or single-site data were common, while prospective external validation in independent settings remained the exception. Within the core charting corpus, 29 (10.5%) records reported no advanced validation feature, whereas external or multicenter validation was charted in 15 (5.5%) records and randomized evaluation in only 16 (5.8%) records. The consequences of this gap are illustrated by the performance degradation of a widely deployed commercial sepsis model when tested across new institutions [36]. Systematic reviews of electronic health record (EHR)–based prediction models similarly report that most evidence derives from retrospective analyses with internal validation, with external validation a relative exception [37-39]. Reviews of machine learning randomized trials further note that many are underpowered and rely on surrogate rather than patient-centered end points [40]. In economic evaluation, the scarcity of prospective assessment means reported returns reflect modeled projections rather than observed real-world outcomes [12,18]. This is not a domain-specific limitation but a field-wide characteristic of how health care AI evidence has been generated.

#### Workflow-Mediated Effectiveness

Across all 4 domains, the studies that demonstrated real-world clinical benefit shared a common feature: the AI output was directly linked to a specific, executable clinical action with minimal decision complexity. The Hypotension Prediction (HYPE) trial succeeded because the model output mapped directly onto an immediately executable anesthesiologist action [41]. Colonoscopy computer-aided detection systems succeeded because the alert appeared in the endoscopist’s visual field at the moment of decision [42-44]. In contrast, sepsis prediction systems requiring interpretation across multiple clinical layers, escalation through care teams, and behavioral change at scale showed more variable results [45-48]. In medication safety, alert fatigue emerged as a recurring failure mode: high-volume, low-specificity alerts erode clinician trust and weaken downstream benefit even when the underlying prediction is accurate [17,29]. Across the corpus, verified workflow or process metrics were reported in only 13 (4.7%) records, underscoring how rarely the behavioral and organizational mechanisms of clinical impact were documented.

#### Infrastructure Fragmentation

Fragmented health data infrastructure was a recurring constraint across all 4 domains, charted in 54 (19.6%) records. Interoperability gaps across EHR systems limited access to diverse training data and impeded model transfer across sites, leaving deployment local and brittle rather than scalable [49]. Transfer learning approaches were frequently proposed as a workaround [50], but these mitigate rather than remove the underlying fragmentation. This barrier corresponds to the inner- and outer-setting constructs of the Consolidated Framework for Implementation Research and the organizational and wider context domains of NASSS as applied to clinical AI [33,51].

#### Equity and Generalizability Deficits

Across the 275-record core charting corpus, explicit equity, fairness, or subgroup performance signals were charted in only 20 (7.3%) records. Evidence that training data imbalances can propagate and amplify existing disparities in clinical care has been documented in the clinical decision support literature [13,14], but equity was rarely examined as a primary outcome in diagnostic AI or predictive analytics studies. In global health contexts, the applicability of models developed in high-resource settings to low- and middle-income environments was identified as an unresolved concern [5]. This gap is consequential: AI systems that perform well on average may perform poorly for underrepresented subgroups, with implications for whether adoption narrows or widens existing health disparities.

#### Economic and Governance Uncertainty

Economic and governance evidence remained underdeveloped relative to technical performance. Among the 53 economic or value assessment records, only 4 (7.5%) were formal economic evaluations, and these were concentrated in European health system settings with heterogeneous analytic perspectives and time horizons. This limits the direct transferability of value claims across health systems and reinforces the need to treat modeled savings or budget effects as context-dependent rather than generalizable evidence of affordability or efficiency. Reported returns frequently omitted implementation and operational costs and, in some cases, reflected revenue expansion rather than genuine efficiency gains [12,18,52]. Hidden downstream costs can also arise when new technologies introduce new error types [53]. On governance, lifecycle or regulatory oversight content was charted in 42 (15.3%) records, pointing to a need for monitoring of performance drift and postdeployment change and for treating clinical AI as an evolving sociotechnical intervention rather than a static product [54,55]. This barrier engages the value proposition and adaptation over time domains of NASSS and the maintenance dimension of RE-AIM as applied to clinical AI [32,34].

**Figure 3.**
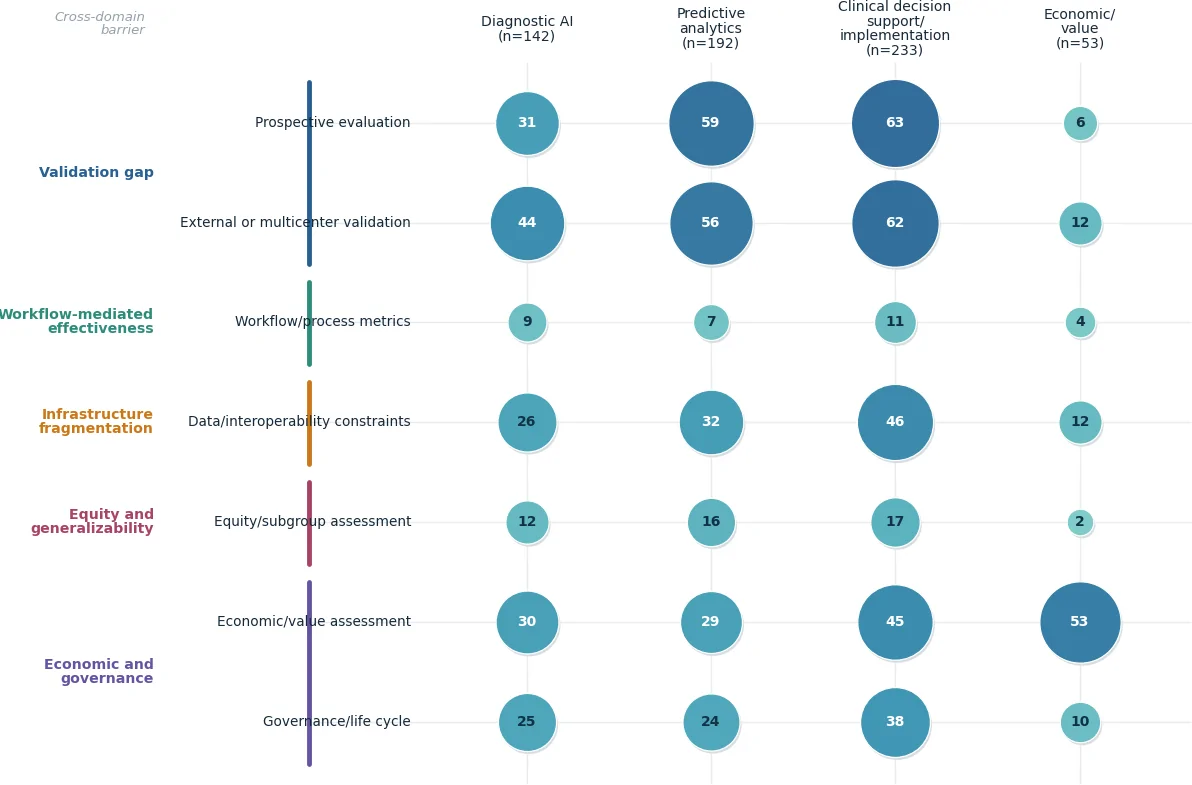
Bubble evidence gap map of the 275-record core charting corpus (2017‐2026), showing the visibility of evidence maturity and barrier-related indicators across the 4 nonmutually coded application domains (diagnostic AI, predictive analytics, clinical decision support or implementation-related applications, and economic or value assessment). Unlike the Sankey diagram in Figure 2, which traces the validation-to-deployment pathway and therefore excludes review or evidence synthesis records that are not applicable to that pathway, this gap map retains the full corpus because review records also carry workflow, equity, infrastructure, and governance indicators relevant to indicator visibility. Bubble size reflects the number of records in which each indicator was charted within a domain. Row and column values should not be summed because records could be coded into more than one domain and could carry more than one indicator. The barrier-related indicators shown here emerged through inductive synthesis of the charted literature and do not represent a formal causal model. The map displays the visibility of indicators in the charted record rather than mutually exclusive study counts or formal quality ratings.

To summarize the descriptive evidence base underlying these barriers, Figure 3 presents a bubble evidence gap map of the 275-record core charting corpus across 4 nonmutually coded application domains and selected evidence maturity indicators. The figure is intended to show the visibility of barrier-related indicators within domains rather than mutually exclusive study counts or formal quality ratings.

**Figure 4.**
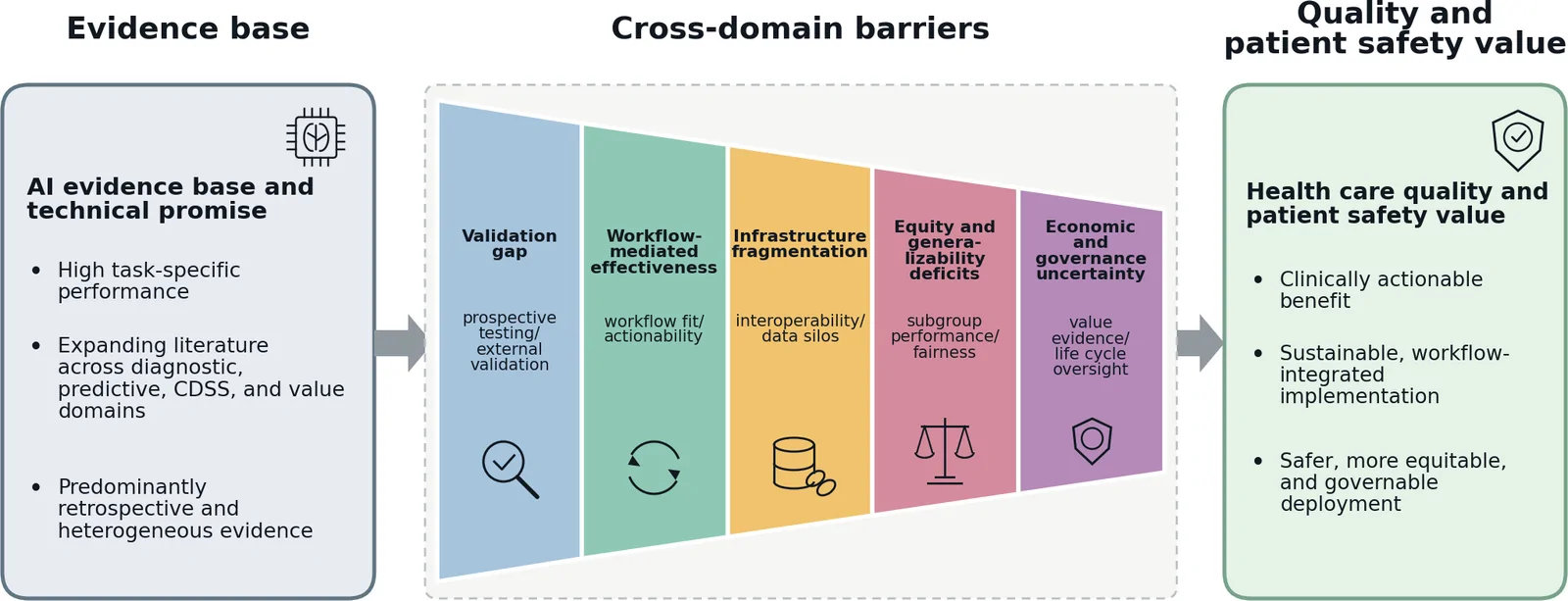
Conceptual synthesis of the 5 cross-domain barriers that may limit translation of health care AI into quality and patient safety value, developed through interpretive synthesis of the 275-record core charting corpus. The left panel represents the technical promise and heterogeneity of the evidence base; the central funnel represents the 5 barriers, namely, the validation gap, workflow-mediated effectiveness, infrastructure fragmentation, equity and generalizability deficits, and economic and governance uncertainty; and the right panel represents the intended end point of responsible AI adoption. This figure is conceptual and should not be interpreted as a causal model, a quantitative evidence map, a formal quality appraisal, or a per-record barrier classification. CDSS: clinical decision support system.

Building on the descriptive evidence gap map in Figure 3, Figure 4 presents an interpretive conceptual synthesis of how the 5 cross-domain barriers may constrain translation from technical promise to quality and patient safety value.

## Discussion

### Principal Findings

This scoping review identifies a persistent gap between the expanding evidence base for health care AI and its translation into quality and patient safety value [4,16]. The descriptive evidence maps showed that the literature spans diagnostic, predictive, clinical decision support, and value assessment domains, but that evidence maturity remains uneven across validation, workflow integration, infrastructure, equity, and governance indicators. Taken together, these findings support a conceptual synthesis of 5 cross-domain barriers to clinical translation. Rather than representing chronological stages, these barriers can constrain translation at multiple points between technical promise and real-world clinical value. The relative importance of these barriers is likely to vary across application domains, health care systems, and implementation settings [51], and the 5-barrier synthesis should therefore be interpreted as a cross-domain organizing framework rather than a uniform ranking that applies in the same way to every context.

### Interpreting the 5 Barriers

#### The Validation Gap

Relatively few of the studies mapped in this review were evaluated prospectively or in external settings, a limitation that is by now documented [37,40]. More informative than its existence, however, is the reason it persists. The field does not lack validation standards. Reporting and risk-of-bias instruments developed specifically for clinical AI, including TRIPOD+AI and the more recent PROBAST+AI (Prediction model Risk of Bias Assessment Tool extension for Artificial Intelligence), already provide detailed guidance for evaluation across translational stages [27,28,56]. These instruments, however, are applied inconsistently and rarely enforced, and machine learning models continue to be published with insufficient sample size justification, inadequate handling of missing data, and limited reporting of performance across subgroups [57,58]. The validation gap thus reflects standards that are available but unimplemented, rather than a genuine absence of methodological consensus.

A second mechanism reinforces this pattern. Strong performance on a retrospective dataset is frequently interpreted as evidence of clinical value; yet, a high accuracy or area under the curve figure provides little assurance that a model improves clinical decisions, reduces avoidable harm, or remains reliable when the patient population shifts [9,10]. The limitations of these measures become apparent when a model is deployed beyond its development setting, where performance can decline substantially and, in one widely cited case, a broadly adopted sepsis model failed on external validation [36]. These findings indicate that progress will require more than an increased number of validation studies. It will require a change in the standard of evidence itself, from discrimination on retrospective data toward prospective demonstration that a model improves care processes and patient outcomes.

#### Workflow-Mediated Effectiveness

An accurate model does not guarantee changes in clinical practice. The implementation literature shows that workflow integration requires more than a technical interface [32,33]. When AI tools fail, the cause is often the clinical environment, not the algorithm [59]. AI outputs may also be introduced without sufficient guidance or training for their use [34], while high-volume, low-relevance alerts are often overridden, contributing to alert fatigue [17]. As a result, these systems conflict with established routines rather than being integrated into them [60,61]. Consequently, technically sound tools are sometimes abandoned after deployment [62]. Workflow fit is not the same as placing a model inside an EHR. It also depends on who receives the output, when it arrives, what action is expected, and whether that action can be completed within existing clinical routines. A system may therefore be technically integrated but clinically ineffective if responsibility is unclear, the output arrives too late, or no feasible action follows.

Three recurring human and organizational factors help explain these implementation failures. The first is interpretability. When clinicians cannot understand how a model reached its conclusion, trust erodes and recommendations are set aside [63]. The second is professional autonomy, as tools perceived to encroach on clinical judgment meet resistance regardless of their accuracy [60]. The third is the lack of behavioral evidence. Across the reviewed literature, indicators such as alert acceptance, override rates, and time to action were rarely recorded. Consequently, the mechanisms that determine whether a model helps or hinders care remain largely unexamined. Without these measures, it is difficult to distinguish failure of the model from failure of the interface, the implementation process, or the clinical pathway expected to act on its output. Workflow-mediated effectiveness is therefore not a secondary refinement to be added after validation. Instead, it is a fundamental requirement for clinical value and currently the least measured aspect in the field.

#### Infrastructure Fragmentation

Fragmented data systems are often treated as a technical problem involving incompatible records and weak interoperability [49]. In this review, infrastructure constraints were charted in 54 (19.6%) records and appeared across all 4 (100%) application domains. Across the charted records, fragmentation took several recurring forms. It surfaced as heterogeneous data structures and clinical terminology, as incomplete integration with EHR systems, and as limited capacity for real-time data flow and monitoring. Interoperability gaps restrict the data available at a site, narrowing the populations on which models are developed and making transfer to other settings less reliable [64]. The result is often a deployment that works locally but is difficult to reproduce or scale.

Model adaptation alone is insufficient to resolve this issue. While techniques such as transfer learning reduce dataset discrepancies [50], they fail to harmonize clinical terminology, build EHR interfaces, or establish pipelines for routine system monitoring. Consequently, infrastructure must be treated as an active component of the intervention rather than a passive background condition. Clinical AI readiness is therefore an organizational challenge as much as a technical one. It depends on whether the required data can be reached and used, whether the system can be connected to existing clinical work, and whether its use and performance can be monitored after deployment.

Reading infrastructure this way also connects it to the other gaps described in this review. Multicenter and external validation depend on data that are comparable across institutions [49], so sites that cannot exchange usable data are unlikely to produce that kind of evidence. Workflow evaluation carries a similar dependency because measures such as alert acceptance or override rates require captured clinician response data [17]. Economic evidence is affected differently. The work of building interfaces, harmonizing data, and supporting users is part of what a system costs in practice, and it is easily left out of an analysis [12]. This review cannot show that fragmentation produced these gaps, although the same limitations plausibly make them harder to close.

Capacity is also unevenly distributed. Institutions with integrated records and dedicated informatics teams can absorb implementation work more readily than community hospitals or resource-constrained health systems, where manual and digital processes may have to run in parallel and where implementation adds directly to clinical workload [65,66]. Settings with substantial need for AI-supported efficiency may therefore be among those least able to adopt and sustain it. Evidence produced in well-resourced settings may also miss the access and representation problems that arise elsewhere. One practical consequence is that studies should describe the infrastructure their systems relied on because readers cannot otherwise judge whether a finding is likely to hold in their own setting. Infrastructure fragmentation therefore does not just delay implementation. It risks concentrating the benefits of health care AI in capacity-rich institutions, which widens existing disparities in care.

#### Equity and Generalizability Deficits

Equity was rarely a primary concern in the studies mapped in this review. That absence should not be interpreted as evidence of equitable performance. It reflects what was examined and reported, not necessarily how these systems performed across populations. When subgroup results are not reported, equal performance has not been demonstrated and remains unknown. The appropriate interpretation is therefore uncertainty rather than confirmed harm. Yet this uncertainty is not reassuring because the pathways through which biases enter clinical AI are already well described [67].

Bias can enter during model design through the choice of outcomes, proxy variables, sampling strategies, and training data [13,14]. For example, using health care cost as a proxy for clinical need caused a widely used algorithm to systematically underestimate the health care needs of Black patients [14]. Such proxies are convenient and may appear reliable when evaluated using aggregate measures, but aggregate performance can conceal important disparities. Intersectional analyses can reveal differences that remain hidden in single-attribute evaluations, including higher underdiagnosis rates for Black women in image-based diagnostics [68]. A model may therefore appear equitable overall while performing poorly for particular patient groups. Technical remedies, including data reweighting and subgroup-specific thresholds, can narrow measured performance gaps, but they cannot fully remove inequities that reflect structural differences embedded in the underlying data [69]. Modifying the algorithm does not resolve the conditions that produced those data.

Generalizability is closely related to equity, but the 2 are not equivalent. A model may retain acceptable average performance in a new hospital while still failing for smaller groups whose outcomes are obscured by the overall estimate. Conversely, similar subgroup accuracy during validation does not guarantee similar clinical benefits because access to the system, clinician responses, and the availability of follow-up care may differ across populations. External validation is commonly framed as a test of whether performance can be reproduced. It should also ask for whom that performance is reproduced and under what conditions of care. This question is especially pressing in global health, where models built in well-resourced systems may not transfer to low- and middle-income settings [5], and a study confined to its development setting cannot adequately answer questions of either generalizability or equity.

Equity is also not settled at deployment. Real-world data change over time, and a model that initially performs similarly across groups may lose that property as patient populations, clinical practices, and patterns of data capture evolve. Without continuous monitoring, such deterioration may remain undetected [70]. Equity is therefore not a fixed attribute that a model permanently retains. It must be assessed before deployment and monitored throughout the operational lifecycle, a standard that the current literature rarely meets. At a minimum, studies should report performance for clinically relevant subgroups together with the demographic and contextual composition of their development and validation cohorts. Equity cannot be meaningfully assessed when the information needed to evaluate it was never collected or reported.

#### Economic and Governance Uncertainty

Economic and governance evaluations of health care AI are often considered separately, yet they share a common limitation. Both are frequently applied as relatively static assessments to technologies and care environments that may continue to change after implementation [70]. Models may be retrained or updated, the data feeding them may shift, and the clinical setting around them may evolve. As a result, the system assessed during initial evaluation, procurement, or approval may no longer fully resemble the system operating in routine care several years later.

Economically, most studies rely on modeled, retrospective, or secondary value evidence [71]. Formal economic evaluations were rare, and the few identified evaluations were context specific, differing by national setting, analytic perspective, and time horizon. More importantly, conventional cost-effectiveness models may not adequately account for AI systems that evolve [19]. These models often omit indirect implementation costs and the expenses of continuous retraining and monitoring. As a result, reported financial returns can overstate the actual value in routine clinical practice [72,73]. Positive economic projections are often just the result of evaluation methods that assume an operational stability these dynamic systems lack. Reported returns may combine efficiency gains, such as reduced staff time, with revenue from downstream procedures, thereby complicating interpretation of the economic case [52].

Governance frameworks show the same limitation from a different angle. Regulatory oversight focuses heavily on premarket approval, while postdeployment monitoring mechanisms remain underdeveloped [70]. Standards for detecting performance drift, recalibrating models, and independent auditing are encouraged but rarely mandated. Existing regulatory frameworks for clinical AI have so far concentrated on premarket evaluation and device-style approval, with comparatively limited mechanisms for continuous postdeployment oversight, which leaves adaptive systems insufficiently governed once in routine use [70,74]. Because of this, adaptive systems can degrade or develop new biases long after initial approval without being detected.

Taken together, these economic and governance gaps reveal a fundamental mismatch between how AI tools are evaluated and how they operate over time [72]. Both forms of assessment can rely heavily on judgments made before or early in routine use, when long-term performance, maintenance requirements, and organizational consequences are still difficult to observe. Addressing this mismatch requires a continuous lifecycle approach rather than reliance on a single premarket approval or initial value assessment point [74].

### A Cross-Cutting Observation: The Behavioral Evidence Gap

Considered together, the 5 barriers converge on a single underlying deficiency. Health care AI is increasingly evaluated for technical performance, but the evidence base remains much thinner on how these systems behave once embedded in clinical work. In this review, that gap was most visible in the scarcity of verified workflow or process indicators, which were charted in only 13 (4.7%) records, but the same absence appeared in other forms. Studies of algorithmic fairness rarely measured how bias interacts with clinician behavior at the point of care, such as alert acceptance or response time, even as model-level disparities were extensively documented [75]. Reviews of clinician trust and adoption identified explainability, professional autonomy, and interface design as important determinants of use, yet these human and organizational mechanisms were seldom quantified in implementation studies [60,63]. The field has therefore accumulated evidence on what models can do under retrospective or controlled conditions, while leaving less well characterized what happens when a model output meets a clinician, a workflow, and a patient. This behavioral evidence gap is not a property of any single barrier. It is a structural feature of how health care AI has been studied, and it helps explain why technical maturity has not consistently translated into demonstrated clinical value.

### Implications for Practice, Policy, and Research

For health systems, the findings imply that AI adoption should be treated as a service-delivery intervention rather than a software procurement decision [65,66]. Model performance should remain necessary, but it is not sufficient for clinical adoption. Before deployment, organizations should require evidence that the model has been evaluated in settings that resemble the intended use environment, that the output is linked to a clear clinical action, and that the local workflow can absorb the intervention without creating new safety burdens. Operationally, this means that AI use cases should begin with stakeholder identification, workflow mapping, and definition of a specific clinical pain point, rather than with model availability alone [61,62]. Health systems should also assess whether they have the local informatics and data science capacity needed for data harmonization, site-specific validation, postdeployment monitoring, drift review, recalibration, and periodic performance review. This standard would shift evaluation from asking whether a model predicts accurately to asking whether it changes care in a measurable, safe, and equitable way.

Regulators and institutional governance bodies face a related task. Current reporting and evaluation frameworks provide increasingly detailed guidance for prediction model development, bias assessment, and clinical AI reporting, yet our synthesis suggests that the main weakness lies in enforcement and lifecycle follow-up rather than in the absence of frameworks [27,28,56-58]. Approval or procurement decisions should therefore include plans for postdeployment monitoring, recalibration, documentation of model updates, and independent audit where feasible. A clinical AI system that changes as data, populations, and workflows change cannot be governed as a static device. It requires recurring evidence that the benefits, risks, and equity effects remain acceptable after implementation [70,74].

Research priorities should also move closer to the point of care. Future studies should report workflow and behavioral end points alongside discrimination metrics, including alert acceptance, override patterns, time to action, clinician workload, escalation behavior, and reasons for nonadherence. Economic evaluations should be conducted alongside real-world implementation whenever possible and should include implementation costs, maintenance costs, retraining, monitoring, clinician time, and downstream resource use [72,73]. Equity evaluation should extend beyond subgroup performance at model launch to include access, adoption, differential response, and performance drift over time. These priorities would make health care AI evaluation more aligned with the outcomes that matter to patients, clinicians, health systems, and regulators.

The cross-domain nature of the findings is itself an implication. Diagnostic AI, predictive analytics, clinical decision support, and value assessment differ in their tasks and evidence traditions, but the same translational constraints recur across them. This convergence supports the use of shared minimum expectations for prospective validation, workflow integration, infrastructure readiness, equity monitoring, and lifecycle governance [58]. Such expectations should not replace domain-specific standards, but they can provide a common floor for responsible AI adoption across health care settings.

### Strengths and Limitations

This review has several strengths. It used an expanded 5-database search strategy across biomedical, multidisciplinary, engineering, nursing, and allied health sources, and the final corpus was supported by a transparent PRISMA flow, complete database search documentation, and a DOI-traceable appendix of included sources. The analysis also moved beyond a single application area by mapping diagnostic AI, predictive analytics, clinical decision support, and economic or value assessment within one synthesis. This made it possible to identify barriers that cut across domains rather than appearing as isolated problems within one clinical specialty. The nonmutual domain coding, Sankey diagram, bubble evidence gap map, and conceptual synthesis figure were designed to make the evidence structure visible without implying formal quality ranking.

Several limitations should be considered when interpreting the findings. First, the review was not prospectively registered, which is a transparency limitation. To mitigate this, the eligibility criteria, staged screening workflow, and charting fields were predefined and reported in full, and audit logs of the search, deduplication, and screening steps were retained to support reproducibility. Second, updated screening and charting were conducted primarily by 1 reviewer, with subset verification and synthesis review by the broader author team. A medical librarian was not formally consulted in developing the search strategy, which may have affected the sensitivity or precision of the search, despite the use of 5 databases, database-specific syntax, PRISMA-S documentation, and retained search audit logs. The reliance on a single primary reviewer may also have influenced study selection and data extraction decisions, particularly for borderline records and interpretive charting fields. To mitigate these risks, the eligibility criteria and charting definitions were predefined, subsets of records were independently checked by 2 additional reviewers, and the complete row-level charting matrix is provided in Multimedia Appendix 3 to support auditability, although these measures cannot fully substitute for independent duplicate screening and duplicate data extraction. Third, the staged screening process used prioritization to manage a very large search yield. Prioritization tiers reflect allocation of review effort rather than confirmed evidence levels, and some potentially relevant records may not have advanced to detailed assessment and charting under the title- and abstract-based prioritization. The findings should therefore be interpreted as characterizing the final 275-record charting corpus rather than all potentially relevant records identified by the search.

A further limitation is that much of the rapid charting relied on titles, abstracts, metadata, and targeted manual verification for selected indicators. As a result, some features that were reported only in full text may have been undercounted. The indicator counts should therefore be interpreted as the visibility of evidence maturity and implementation signals in the charted record, not as definitive judgments about study conduct. This is particularly important for workflow metrics, equity assessment, governance content, and infrastructure constraints, which are often described inconsistently across publications.

The review was also limited to English-language records. This restriction may have excluded relevant studies from non-English health systems, particularly in countries with substantial AI development and clinical deployment activity, and it may underrepresent local implementation reports, regulatory experience, and health system learning published outside English-language indexed journals. This affects the global generalizability of the evidence map. The review was designed, however, to map peer-reviewed, indexed literature on AI for health care quality and patient safety, not patent activity, commercial innovation, or all regional deployments, and we therefore interpret the findings as applying to the English-language indexed evidence base. To reduce the risk of missing relevant peer-reviewed evidence within that scope, we searched 5 complementary databases spanning biomedical, multidisciplinary, engineering, nursing, allied health, and health technology literature, without geographic restriction.

However, the search did not include supplementary methods such as reference list scanning, forward citation chasing, expert referral, or searching of the International HTA Database, so some eligible studies indexed outside the 5 databases, particularly economic or health technology assessment reports, may have been missed; the findings on economic evaluation should therefore be read as describing the charted corpus rather than as an exhaustive account of that literature. The breadth of the review was also a deliberate trade-off since mapping across 4 domains necessarily limited the depth of clinical detail available for any single specialty, disease area, or AI modality. Finally, consistent with scoping review methodology, we did not conduct a formal risk-of-bias appraisal or meta-analysis, and the findings should be read as a descriptive and interpretive evidence map rather than as a pooled estimate of effectiveness.

### Conclusions

Health care AI evidence is broad, rapidly expanding, and increasingly visible across diagnostic, predictive, decision support, and value applications. The central finding of this review is that the binding constraint on clinical value is no longer primarily technical. Translation is limited instead by a set of structural barriers, namely weak prospective and external validation, insufficient measurement of workflow-mediated effectiveness, fragmented data infrastructure, underdeveloped equity and generalizability assessment, and uncertainty about economic value and lifecycle governance. This review differs from existing work in both scope and approach. Whereas most reviews concentrate on a single application area, it maps evidence across 4 domains within one synthesis, which is what makes the recurrence of the same barriers across domains visible. Its methodological contribution is to anchor this synthesis in the WHO quality of care dimensions and to keep descriptive charting separate from interpretive synthesis, so that the derivation of the barriers remains traceable. Its conceptual contribution is to reframe the central problem of health care AI as structural rather than technical. For health care AI to improve quality and patient safety at scale, evaluation standards need to move closer to the realities of clinical use. Future evidence should demonstrate not only that a model performs well but also that it works across settings, supports executable clinical action, remains safe over time, reduces rather than amplifies inequity, and produces value once implementation costs and governance requirements are considered. Responsible adoption should therefore treat prospective validation, workflow-integrated deployment, infrastructure readiness, equity monitoring, and lifecycle governance as core requirements rather than optional additions.

## Supplementary material

10.2196/95157 Multimedia Appendix 1 Five-database search strategy, export audit, deduplication, and screening flow.

10.2196/95157 Multimedia Appendix 2 Operational rules and record-level dispositions for the staged screening and prioritization workflow.

10.2196/95157 Multimedia Appendix 3 Full row-level charting matrix for the 275-record core corpus, including application domain coding, evidence maturity fields, barrier indicators, economic evaluation fields, and count check documentation.

10.2196/95157 Multimedia Appendix 4 Illustrative primary and secondary evidence across application domains.

10.2196/95157 Checklist 1 PRISMA-ScR checklist.

## Abbreviations

EHR: electronic health record
HYPE: Hypotension Prediction
NASSS: nonadoption, abandonment, scale-up, spread, and sustainability
PRISMA: Preferred Reporting Items for Systematic Reviews and Meta-Analyses
PRISMA-S: Preferred Reporting Items for Systematic Reviews and Meta-Analyses literature search extension
PRISMA-ScR: Preferred Reporting Items for Systematic Reviews and Meta-Analyses extension for Scoping Reviews
PROBAST: Prediction Model Risk of Bias Assessment Tool
PROBAST+AI: Prediction Model Risk of Bias Assessment Tool extension for Artificial Intelligence
RE-AIM: reach, effectiveness, adoption, implementation, and maintenance
TRIPOD+AI: Transparent Reporting of a Multivariable Prediction Model for Individual Prognosis or Diagnosis extension for Artificial Intelligence
WHO: World Health Organization

## References

[1] Hyland SL, Faltys M, Hüser M (2020). Early prediction of circulatory failure in the intensive care unit using machine learning. Nat Med.

[2] Acosta JN, Falcone GJ, Rajpurkar P, Topol EJ (2022). Multimodal biomedical AI. Nat Med.

[3] Topol EJ (2019). High-performance medicine: the convergence of human and artificial intelligence. Nat Med.

[4] Kelly CJ, Karthikesalingam A, Suleyman M, Corrado G, King D (2019). Key challenges for delivering clinical impact with artificial intelligence. BMC Med.

[5] Schwalbe N, Wahl B (2020). Artificial intelligence and the future of global health. The Lancet.

[6] Silcox C, Zimlichmann E, Huber K (2024). The potential for artificial intelligence to transform healthcare: perspectives from international health leaders. NPJ Digit Med.

[7] Davenport T, Kalakota R (2019). The potential for artificial intelligence in healthcare. Future Healthc J.

[8] Vaishya R, Javaid M, Khan IH, Vaish A, Iyengar KP (2021). Significant role of modern technologies for COVID-19 pandemic. J Ind Intg Mgmt.

[9] Liu X, Faes L, Kale AU (2019). A comparison of deep learning performance against health-care professionals in detecting diseases from medical imaging: a systematic review and meta-analysis. Lancet Digit Health.

[10] Nagendran M, Chen Y, Lovejoy CA (2020). Artificial intelligence versus clinicians: systematic review of design, reporting standards, and claims of deep learning studies. BMJ.

[11] Fleuren LM, Klausch TLT, Zwager CL (2020). Machine learning for the prediction of sepsis: a systematic review and meta-analysis of diagnostic test accuracy. Intensive Care Med.

[12] Wolff J, Pauling J, Keck A, Baumbach J (2020). The economic impact of artificial intelligence in health care: systematic review. J Med Internet Res.

[13] Gianfrancesco MA, Tamang S, Yazdany J, Schmajuk G (2018). Potential biases in machine learning algorithms using electronic health record data. JAMA Intern Med.

[14] Obermeyer Z, Powers B, Vogeli C, Mullainathan S (2019). Dissecting racial bias in an algorithm used to manage the health of populations. Science.

[15] McGenity C, Clarke EL, Jennings C (2024). Artificial intelligence in digital pathology: a systematic review and meta-analysis of diagnostic test accuracy. npj Digit Med.

[16] Bates DW, Levine D, Syrowatka A (2021). The potential of artificial intelligence to improve patient safety: a scoping review. npj Digit Med.

[17] Graafsma J, Murphy RM, van de Garde EMW (2024). The use of artificial intelligence to optimize medication alerts generated by clinical decision support systems: a scoping review. J Am Med Inform Assoc.

[18] Kastrup N, Holst-Kristensen AW, Valentin JB (2024). Landscape and challenges in economic evaluations of artificial intelligence in healthcare: a systematic review of methodology. BMC Digit Health.

[19] Vithlani J, Hawksworth C, Elvidge J, Ayiku L, Dawoud D (2023). Economic evaluations of artificial intelligence-based healthcare interventions: a systematic literature review of best practices in their conduct and reporting. Front Pharmacol.

[20] Kruk ME, Gage AD, Arsenault C (2018). High-quality health systems in the Sustainable Development Goals era: time for a revolution. Lancet Glob Health.

[21] Tunçalp Ӧ., Were WM, MacLennan C (2015). Quality of care for pregnant women and newborns-the WHO vision. BJOG.

[22] Peters M, Godfrey C, McInerney P, Munn Z, Tricco A, Khalil H (2019). JBI Reviewer’s Manual.

[23] Tricco AC, Lillie E, Zarin W (2018). PRISMA Extension for Scoping Reviews (PRISMA-ScR): checklist and explanation. Ann Intern Med.

[24] Peters MDJ, Marnie C, Tricco AC (2020). Updated methodological guidance for the conduct of scoping reviews. JBI Evidence Synthesis.

[25] Peters MDJ, Marnie C, Colquhoun H (2021). Scoping reviews: reinforcing and advancing the methodology and application. Syst Rev.

[26] Rethlefsen ML, Kirtley S, Waffenschmidt S (2021). PRISMA-S: an extension to the PRISMA Statement for Reporting Literature Searches in Systematic Reviews. Syst Rev.

[27] Collins GS, Moons KGM, Dhiman P (2024). TRIPOD+AI statement: updated guidance for reporting clinical prediction models that use regression or machine learning methods. BMJ.

[28] Wolff RF, Moons KGM, Riley RD (2019). PROBAST: a tool to assess the risk of bias and applicability of prediction model studies. Ann Intern Med.

[29] Choudhury A, Asan O (2020). Role of artificial intelligence in patient safety outcomes: systematic literature review. JMIR Med Inform.

[30] Brunton G, Oliver S, Thomas J (2020). Innovations in framework synthesis as a systematic review method. Res Synth Methods.

[31] Kastner M, Tricco AC, Soobiah C (2012). What is the most appropriate knowledge synthesis method to conduct a review? Protocol for a scoping review. BMC Med Res Methodol.

[32] Shin HD, Hamovitch E, Gatov E (2025). The NASSS (non-adoption, abandonment, scale-up, spread and sustainability) framework use over time: a scoping review. PLOS Digit Health.

[33] Gama F, Tyskbo D, Nygren J, Barlow J, Reed J, Svedberg P (2022). Implementation frameworks for artificial intelligence translation into health care practice: scoping review. J Med Internet Res.

[34] Finkelstein J, Gabriel A, Schmer S, Truong TT, Dunn A (2024). Identifying facilitators and barriers to implementation of AI-assisted clinical decision support in an electronic health record system. J Med Syst.

[35] Creative commons attribution 4.0 international (CC BY 4.0). Creative Commons.

[36] Wong A, Otles E, Donnelly JP (2021). External validation of a widely implemented proprietary sepsis prediction model in hospitalized patients. JAMA Intern Med.

[37] Yasrebi-de Kom IAR, Dongelmans DA, de Keizer NF (2023). Electronic health record-based prediction models for in-hospital adverse drug event diagnosis or prognosis: a systematic review. J Am Med Inform Assoc.

[38] Baddal B, Taner F, Uzun Ozsahin D (2024). Harnessing of artificial intelligence for the diagnosis and prevention of hospital-acquired infections: a systematic review. Diagnostics (Basel).

[39] El Arab RA, Almoosa Z, Alkhunaizi M, Abuadas FH, Somerville J (2025). Artificial intelligence in hospital infection prevention: an integrative review. Front Public Health.

[40] Plana D, Shung DL, Grimshaw AA, Saraf A, Sung JJY, Kann BH (2022). Randomized clinical trials of machine learning interventions in health care: a systematic review. JAMA Netw Open.

[41] Wijnberge M, Geerts BF, Hol L (2020). Effect of a machine learning-derived early warning system for intraoperative hypotension vs standard care on depth and duration of intraoperative hypotension during elective noncardiac surgery: the HYPE randomized clinical trial. JAMA.

[42] Gong D, Wu L, Zhang J (2020). Detection of colorectal adenomas with a real-time computer-aided system (ENDOANGEL): a randomised controlled study. Lancet Gastroenterol Hepatol.

[43] Wang P, Liu X, Berzin TM (2020). Effect of a deep-learning computer-aided detection system on adenoma detection during colonoscopy (CADe-DB trial): a double-blind randomised study. Lancet Gastroenterol Hepatol.

[44] Barua I, Wieszczy P, Kudo SE (2022). Real-time artificial intelligence-based optical diagnosis of neoplastic polyps during colonoscopy. NEJM Evid.

[45] Adams R, Henry KE, Sridharan A (2022). Prospective, multi-site study of patient outcomes after implementation of the TREWS machine learning-based early warning system for sepsis. Nat Med.

[46] Boussina A, Shashikumar SP, Malhotra A (2024). Impact of a deep learning sepsis prediction model on quality of care and survival. NPJ Digit Med.

[47] Shimabukuro DW, Barton CW, Feldman MD, Mataraso SJ, Das R (2017). Effect of a machine learning-based severe sepsis prediction algorithm on patient survival and hospital length of stay: a randomised clinical trial. BMJ Open Respir Res.

[48] Persson I, Macura A, Becedas D, Sjövall F (2024). Early prediction of sepsis in intensive care patients using the machine learning algorithm NAVOY® Sepsis, a prospective randomized clinical validation study. J Crit Care.

[49] Mandl KD, Gottlieb D, Mandel JC (2024). Integration of AI in healthcare requires an interoperable digital data ecosystem. Nat Med.

[50] Katsamenis I, Protopapadakis E, Voulodimos A, Doulamis A, Doulamis N Transfer learning for COVID-19 pneumonia detection and classification in chest x-ray images.

[51] Alami H, Lehoux P, Papoutsi C, Shaw SE, Fleet R, Fortin JP (2024). Understanding the integration of artificial intelligence in healthcare organisations and systems through the NASSS framework: a qualitative study in a leading Canadian academic centre. BMC Health Serv Res.

[52] Bharadwaj P, Nicola L, Breau-Brunel M (2024). Unlocking the value: quantifying the return on investment of hospital artificial intelligence. J Am Coll Radiol.

[53] Zhou L, Blackley SV, Kowalski L (2018). Analysis of errors in dictated clinical documents assisted by speech recognition software and professional transcriptionists. JAMA Netw Open.

[54] Beam AL, Drazen JM, Kohane IS, Leong TY, Manrai AK, Rubin EJ (2023). Artificial intelligence in medicine. N Engl J Med.

[55] Goldberg CB, Adams L, Blumenthal D (2024). To do no harm - and the most good - with AI in health care. Nat Med.

[56] Moons KGM, Damen JAA, Kaul T (2025). PROBAST+AI: an updated quality, risk of bias, and applicability assessment tool for prediction models using regression or artificial intelligence methods. BMJ.

[57] Andaur Navarro CL, Damen JAA, Takada T (2021). Risk of bias in studies on prediction models developed using supervised machine learning techniques: systematic review. BMJ.

[58] Crossnohere NL, Elsaid M, Paskett J, Bose-Brill S, Bridges JFP (2022). Guidelines for artificial intelligence in medicine: literature review and content analysis of frameworks. J Med Internet Res.

[59] Yu F, Moehring A, Banerjee O, Salz T, Agarwal N, Rajpurkar P (2024). Heterogeneity and predictors of the effects of AI assistance on radiologists. Nat Med.

[60] Lambert SI, Madi M, Sopka S (2023). An integrative review on the acceptance of artificial intelligence among healthcare professionals in hospitals. npj Digit Med.

[61] Strohm L, Hehakaya C, Ranschaert ER, Boon WPC, Moors EHM (2020). Implementation of artificial intelligence (AI) applications in radiology: hindering and facilitating factors. Eur Radiol.

[62] Leenen JPL, Hiemstra P, Ten Hoeve MM (2025). Exploring the complex nature of implementation of artificial intelligence in clinical practice: an interview study with healthcare professionals, researchers and policy and governance experts. PLOS Digit Health.

[63] Abbas Q, Jeong W, Lee SW (2025). Explainable AI in clinical decision support systems: a meta-analysis of methods, applications, and usability challenges. Healthcare (Basel).

[64] Chomutare T, Tejedor M, Svenning TO (2022). Artificial intelligence implementation in healthcare: a theory-based scoping review of barriers and facilitators. Int J Environ Res Public Health.

[65] Nair M, Svedberg P, Larsson I, Nygren JM (2024). A comprehensive overview of barriers and strategies for AI implementation in healthcare: mixed-method design. PLOS ONE.

[66] Qi Y, Mohamad E, Azlan AA, Zhang C (2025). Utilization of artificial intelligence in clinical practice: a systematic review of China’s experiences. Digit Health.

[67] The Lancet (2023). AI in medicine: creating a safe and equitable future. The Lancet.

[68] Yang Y, Liu Y, Liu X (2025). Demographic bias of expert-level vision-language foundation models in medical imaging. Sci Adv.

[69] Huang J, Galal G, Etemadi M, Vaidyanathan M (2022). Evaluation and mitigation of racial bias in clinical machine learning models: scoping review. JMIR Med Inform.

[70] Goktas P, Grzybowski A (2025). Shaping the future of healthcare: ethical clinical challenges and pathways to trustworthy AI. J Clin Med.

[71] Sahni NR, Stein G, Zemmel R, Cutler DM, Agrawal A, Gans J, Goldfarb A, Tucker CE (2024). The Economics of Artificial Intelligence: Health Care Challenges.

[72] Zhang Y, Wang L, Liang Y (2026). A systematic review of the methods and quality of economic evaluations for artificial-intelligence-assisted cancer screening or diagnosis. Value Health.

[73] El Arab RA, Al Moosa OA (2025). Systematic review of cost effectiveness and budget impact of artificial intelligence in healthcare. NPJ Digit Med.

[74] Labkoff S, Oladimeji B, Kannry J (2024). Toward a responsible future: recommendations for AI-enabled clinical decision support. J Am Med Inform Assoc.

[75] Goh E, Bunning B, Khoong EC (2025). Physician clinical decision modification and bias assessment in a randomized controlled trial of AI assistance. Commun Med (Lond).

