# Supplementary material for: AI for Health Care Quality and Patient Safety: Scoping Review of Diagnostic, Predictive, and Decision Support Applications
Source: J Med Internet Res. 2026 Sep 21;28:e95157. doi: 10.2196/95157 (PMC13592239; doi:10.2196/95157)
Supplement: Supplementary file 1 [file jmir-v28-e95157-s001.docx]

**Multimedia Appendix 1. Five-Database Search Strategy, Export Audit, Deduplication, and Screening Flow**

# 1. Purpose and scope

This appendix documents the five-database search, the export and import audit trail, the local deduplication workflow, the staged screening workflow, and the traceability of the final core charting corpus. It is provided to support transparent reporting of the search strategy in line with the PRISMA-S extension and to allow each search component to be reproduced. Searches were executed on 2026-05-18 and covered records published from January 1, 2017, to April 30, 2026.

# 2. Databases searched and record counts before deduplication

| **Database** | **Platform** | **Search field/mode** | **Date range** | **Language** | **Document/content type** | **Hits** |
| --- | --- | --- | --- | --- | --- | --- |
| PubMed/MEDLINE | PubMed | MeSH Major Topic + title/abstract | 2017-01-01 to 2026-04-30 | English | Editorials, comments, letters, news, and animal-only records are excluded by the filter | 16,561 |
| Scopus | Scopus | TITLE-ABS-KEY | 2017-01-01 to 2026-04-30 | English | Article + Review (main) and Conference Paper (supplementary) | 15,145 |
| Web of Science Core Collection | Web of Science | Topic Search (TS) | 2017-01-01 to 2026-04-30 | English | Article + Review | 7,163 |
| IEEE Xplore | IEEE Xplore | Document Title + Abstract | 2017-01-01 to 2026-04-30 | English | Conferences, Journals, Early Access; books/magazines/standards/courses excluded | 3,141 |
| CINAHL Plus with Full Text | EBSCOhost | Title + Abstract | 2017-01-01 to 2026-04-30 | English | No source-type restriction at the search stage | 1,384 |
| Total |  |  |  |  |  | 43,394 |

# 3. Publication-type filtering across databases

Publication-type exclusions were implemented differently across databases because publication-type indexing and export fields are not equivalent across platforms. PubMed/MEDLINE provides controlled publication-type tags, so editorials, comments, letters, and news records were excluded directly in the PubMed query. In Scopus, Web of Science, IEEE Xplore, and CINAHL, equivalent publication-type exclusions were not applied at the search-string stage because field definitions and indexing were less consistent across platforms. Instead, noneligible publication types were handled uniformly during the staged screening workflow, which excluded editorials, commentaries, letters, news items, books, errata, retracted publications, and short conference abstracts without sufficient methods or results.

# 4. Supplementary search sources

Forward-citation chasing and the International HTA Database were considered as supplementary search sources but were not implemented as logged formal search sources for this review. They are therefore not described as part of the formal five-database search corpus. Background and consensus references identified outside the formal search were used only for contextual discussion and are distinguished from the charted evidence corpus throughout the manuscript.

# 5. Export and import audit

Identified-record counts for the PRISMA flow use database hit counts. Local deduplication used parsed and imported records. The only retained discrepancy between identified and parsed records was a documented 37-record difference in the Scopus main-search export and import.

| **Database/source** | **Hits** | **Parsed/imported** | **Difference** | **Notes** |
| --- | --- | --- | --- | --- |
| PubMed/MEDLINE | 16,561 | 16,561 | 0 | Exported in three non-overlapping segments due to export size limits. |
| Scopus | 15,145 | 15,108 | -37 | Main article/review search imported 37 fewer records than database hits; discrepancy retained in audit log. |
| Web of Science | 7,163 | 7,163 | 0 | Database hit count used for PRISMA identification. |
| IEEE Xplore | 3,141 | 3,141 | 0 | RIS citation export with citation and abstract fields when available. |
| CINAHL Plus with Full Text | 1,384 | 1,384 | 0 | EBSCOhost full-result RIS export. |
| Total | 43,394 | 43,357 | -37 | The 37-record difference corresponds to the Scopus export/import discrepancy. |

# 6. Database-specific export segmentation

**6.1 PubMed/MEDLINE export segments**

| **Segment** | **History query** | **Hits** | **Export file** |
| --- | --- | --- | --- |
| 1 | #1 AND (2017/01/01 : 2021/12/31) | 4,964 | PubMed_MEDLINE_2017_2021.nbib |
| 2 | #3 NOT #2 | 5,334 | PubMed_MEDLINE_2022_2024_nooverlap.nbib |
| 3 | #4 NOT (#2 OR #5) | 6,263 | PubMed_MEDLINE_2025_2026_JanApr_nooverlap.nbib |
| Total |  | 16,561 | Segment total equals full-range hit count |

**6.2 Scopus export batches**

| **Batch** | **Document type** | **Year(s)** | **Hits** |
| --- | --- | --- | --- |
| Main 2017 | Article; Review | 2017 | 196 |
| Main 2018 | Article; Review | 2018 | 307 |
| Main 2019 | Article; Review | 2019 | 446 |
| Main 2020 | Article; Review | 2020 | 626 |
| Main 2021 | Article; Review | 2021 | 791 |
| Main 2022 | Article; Review | 2022 | 970 |
| Main 2023 | Article; Review | 2023 | 1,212 |
| Main 2024 | Article; Review | 2024 | 1,781 |
| Main 2025 | Article; Review | 2025 | 3,202 |
| Main 2026 | Article; Review | 2026 | 2,025 |
| Main total | Article; Review | 2017- 2026 | 11,556 |
| CP supplementary | Conference paper | 2017- 2026 | 3,589 |
| Scopus total | Article; Review; Conference paper | 2017- 2026 | 15,145 |

**6.3 Web of Science export batches**

| **Year** | **Hits** | **Year** | **Hits** |
| --- | --- | --- | --- |
| 2017 | 81 | 2022 | 613 |
| 2018 | 131 | 2023 | 759 |
| 2019 | 200 | 2024 | 1,019 |
| 2020 | 325 | 2025 | 2,288 |
| 2021 | 492 | 2026 | 1,255 |
| Total |  |  | 7,163 |

**6.4 IEEE Xplore and CINAHL export notes**

IEEE Xplore was exported as RIS citation files with citation and abstract fields when available. The final post-filter result count and the imported count were both 3,141. CINAHL Plus with Full Text was exported as a single RIS file using the EBSCOhost full-result export; the database hit count and imported count were both 1,384.

# 7. Complete search strategies

**7.1 PubMed/MEDLINE (search date 2026-05-18)**

( ( "Artificial Intelligence"[Majr] OR "Machine Learning"[Majr] OR

"Deep Learning"[Majr] OR "Neural Networks, Computer"[Majr] OR

"artificial intelligence"[tiab] OR "machine learning"[tiab] OR

"deep learning"[tiab] OR neural network*[tiab] OR

"large language model"[tiab] OR "large language models"[tiab] OR

"foundation model"[tiab] OR "foundation models"[tiab] )

AND

( "Quality of Health Care"[Majr] OR "Patient Safety"[Majr] OR

"Decision Support Systems, Clinical"[Majr] OR

"patient safety"[tiab] OR "quality of care"[tiab] OR

"healthcare quality"[tiab] OR "health care quality"[tiab] OR

"clinical decision support"[tiab] OR "medical error"[tiab] OR

"medical errors"[tiab] ) )

AND ("2017/01/01"[dp] : "2026/04/30"[dp])

AND english[la]

NOT (animals[mh] NOT humans[mh])

NOT (editorial[pt] OR comment[pt] OR letter[pt] OR news[pt])

**7.2 Scopus main search (Article + Review)**

TITLE-ABS-KEY (

( "artificial intelligence" OR "machine learning" OR "deep learning" OR

"neural network" OR "neural networks" OR "large language model" OR

"large language models" OR "foundation model" OR "foundation models" )

AND

( "patient safety" OR "quality of care" OR "healthcare quality" OR

"health care quality" OR "clinical decision support" OR

"medical error" OR "medical errors" OR "medication error" OR

"medication errors" ) )

AND PUBYEAR > 2016 AND PUBYEAR < 2027

AND ( LIMIT-TO ( DOCTYPE , "ar" ) OR LIMIT-TO ( DOCTYPE , "re" ) )

AND LIMIT-TO ( LANGUAGE, "English" )

**7.3 Scopus supplementary search (Conference Paper)**

TITLE-ABS-KEY (

( "artificial intelligence" OR "machine learning" OR "deep learning" OR

"neural network" OR "neural networks" OR "large language model" OR

"large language models" OR "foundation model" OR "foundation models" )

AND

( "patient safety" OR "quality of care" OR "healthcare quality" OR

"health care quality" OR "clinical decision support" OR

"medical error" OR "medical errors" OR "medication error" OR

"medication errors" ) )

AND PUBYEAR > 2016 AND PUBYEAR < 2027

AND LIMIT-TO ( DOCTYPE , "cp" )

AND LIMIT-TO ( LANGUAGE , "English" )

**7.4 Web of Science Core Collection**

TS=(

( "artificial intelligence" OR "machine learning" OR "deep learning" OR

"neural network*" OR "large language model*" OR "foundation model*" )

AND

( "patient safety" OR "quality of care" OR "healthcare quality" OR

"health care quality" OR "clinical decision support" OR

"medical error*" OR "medication error*" ) )

AND PY=(2017-2026)

AND LA=(English)

**7.5 IEEE Xplore**

( ("Document Title":"artificial intelligence") OR ("Document Title":"machine learning")

OR ("Document Title":"deep learning") OR ("Document Title":"neural network")

OR ("Document Title":"neural networks") OR ("Document Title":"large language model")

OR ("Document Title":"large language models") OR ("Document Title":"foundation model")

OR ("Document Title":"foundation models") OR ("Document Title":"AI-assisted")

OR ("Document Title":"AI-enabled") OR ("Document Title":"AI-based")

OR ("Abstract":"artificial intelligence") OR ("Abstract":"machine learning")

OR ("Abstract":"deep learning") OR ("Abstract":"neural network")

OR ("Abstract":"neural networks") OR ("Abstract":"large language model")

OR ("Abstract":"large language models") OR ("Abstract":"foundation model")

OR ("Abstract":"foundation models") OR ("Abstract":"AI-assisted")

OR ("Abstract":"AI-enabled") OR ("Abstract":"AI-based") )

AND

( ("Document Title":"patient safety") OR ("Document Title":"quality of care")

OR ("Document Title":"healthcare quality") OR ("Document Title":"health care quality")

OR ("Document Title":"clinical decision support") OR ("Document Title":"decision support system")

OR ("Document Title":"decision support systems") OR ("Document Title":"medical error")

OR ("Document Title":"medical errors") OR ("Document Title":"medication error")

OR ("Document Title":"medication errors") OR ("Abstract":"patient safety")

OR ("Abstract":"quality of care") OR ("Abstract":"healthcare quality")

OR ("Abstract":"health care quality") OR ("Abstract":"clinical decision support")

OR ("Abstract":"decision support system") OR ("Abstract":"decision support systems")

OR ("Abstract":"medical error") OR ("Abstract":"medical errors")

OR ("Abstract":"medication error") OR ("Abstract":"medication errors") )

Filters: Publication years 2017-2026; Content types Conferences, Journals,

Early Access Articles; excluded Books, Magazines, Standards, Courses.

**7.6 CINAHL Plus with Full Text (EBSCOhost)**

( TI ( "artificial intelligence" OR "machine learning" OR "deep learning" OR

"neural network" OR "neural networks" OR "large language model" OR

"large language models" OR "foundation model" OR "foundation models" OR

"AI-assisted" OR "AI assisted" OR "AI-enabled" OR "AI enabled" OR

"AI-based" OR "AI based" )

OR AB ( "artificial intelligence" OR "machine learning" OR "deep learning" OR

"neural network" OR "neural networks" OR "large language model" OR

"large language models" OR "foundation model" OR "foundation models" OR

"AI-assisted" OR "AI assisted" OR "AI-enabled" OR "AI enabled" OR

"AI-based" OR "AI based" ) )

AND

( TI ( "patient safety" OR "quality of care" OR "healthcare quality" OR

"health care quality" OR "clinical decision support" OR

"decision support system" OR "decision support systems" OR

"medical error" OR "medical errors" OR "medication error" OR

"medication errors" )

OR AB ( "patient safety" OR "quality of care" OR "healthcare quality" OR

"health care quality" OR "clinical decision support" OR

"decision support system" OR "decision support systems" OR

"medical error" OR "medical errors" OR "medication error" OR

"medication errors" ) )

Filters: Publication date January 2017 to April 2026; English Language.

# 8. Deduplication workflow

Raw database exports were parsed locally and deduplicated using a conservative Python workflow. Fuzzy matching was disabled to minimize false duplicate merges, especially between conference versions, journal extensions, protocols, and related studies with similar titles. Duplicate-union rules were applied in the following order.

| **Rule order** | **Conservative duplicate-union rule** |
| --- | --- |
| 1 | Exact PMID match |
| 2 | Exact DOI match |
| 3 | Normalized title + year + first-author exact match |
| 4 | Normalized title + year + journal exact match |
| 5 | Long normalized title + year exact match |

| **Source database** | **Parsed input** | **Retained after deduplication** | **Removed as duplicates** |
| --- | --- | --- | --- |
| CINAHL | 1,384 | 327 | 1,057 |
| IEEE Xplore | 3,141 | 2,897 | 244 |
| PubMed/MEDLINE | 16,561 | 16,029 | 532 |
| Scopus | 15,108 | 7,969 | 7,139 |
| Web of Science | 7,163 | 443 | 6,720 |
| Total | 43,357 | 27,665 | 15,692 |

# 9. Staged screening and evidence-prioritization flow

After deduplication, records underwent a staged deterministic and manual screening workflow. Deterministic rules removed records that clearly failed predefined eligibility criteria. Remaining records were prioritized by domain-signal thresholds, manually screened in the high-signal pool, and then prioritized for detailed charting using Stage B evidence-feature prioritization and a final title and abstract based charting prioritization. The staged prioritization supported a focused scoping review under time constraints; the counts represent a staged screening and evidence-prioritization workflow rather than independent dual screening of all 27,665 deduplicated records.

| **Stage** | **Count** | **Notes/formula** |
| --- | --- | --- |
| Records identified from five databases | 43,394 | Sum of database hit counts before deduplication |
| Records parsed/imported for deduplication | 43,357 | 37 fewer than hits (Scopus export/import discrepancy) |
| Duplicate records removed | 15,692 | Conservative local deduplication |
| Records after deduplication (screened) | 27,665 | 43,357 - 15,692 |
| Excluded by deterministic rule-based criteria | 10,246 | Clearly ineligible publication types, short abstracts, non-healthcare/technical records |
| Not advanced after the Stage A scope-signal threshold | 16,041 | Matched fewer than 3 scope-signal groups; retained in the audit trail |
| High-signal pool manually screened | 1,378 | 27,665 - 10,246 - 16,041 |
| Excluded at title/abstract screening | 96 | Manual title/abstract exclusion |
| Retained for Stage B evidence-feature prioritization | 1,282 | 1,378 - 96 |
| Tier A records advanced to the final title and abstract based charting prioritization | 377 | Stage B Tier A selection |
| Not advanced after Stage B evidence-feature prioritization | 905 | 1,282 - 377; Retained in the audit trail |
| Excluded on eligibility grounds at the final charting prioritization | 2 | 1 with no eligible AI or machine-learning focus; 1 nonhuman or veterinary healthcare context |
| Not advanced at the final charting prioritization | 99 | Composite score below the archived threshold without an explicit economic or value signal |
| Reports advanced to report-level assessment and charting | 276 | 377 - 99 - 2 |
| Duplicate report removed at final reconciliation | 1 | Documented in Multimedia Appendix 3 |
| Retained in core charting corpus | 275 | 276 - 1 |

*Operational rules, signal dictionaries, thresholds, scoring weights, and the record-level audit trail for the staged prioritization are provided in Multimedia Appendix 5.*

# 10. Eligibility criteria applied during staged screening

Records were eligible when they involved human health-care settings, evaluated or reviewed AI or machine-learning approaches, and addressed at least one review domain (diagnostic AI, predictive analytics or early warning, clinical decision support, or economic or value assessment) relevant to health-care quality and patient safety. Full-length journal articles, reviews, systematic or scoping reviews, meta-analyses, economic evaluations, validation studies, implementation studies, and full conference papers with sufficient methods and results were eligible. Editorials, commentaries, opinion pieces, letters, news items, books and book chapters, standards, courses, protocols without results, short conference abstracts, records without sufficient methods or results, retracted publications, non-English records, animal-only or nonhuman studies, non-health-care AI applications, and purely technical AI studies without clinical relevance were excluded or not advanced.

# 11. Core corpus and traceability

The final core charting corpus contains 275 records. Application domains were coded non-mutually, because a single record could address more than one domain, such as predictive analytics embedded within clinical decision support. The four domain counts are non-mutually exclusive and should not be summed. Record-level metadata with DOI and PMID traceability is provided in Multimedia Appendix 3.

| **Non-mutually exclusive application domain (275 records)** | **n** |
| --- | --- |
| Diagnostic AI | 142 |
| Predictive analytics | 192 |
| Clinical decision support / implementation-related applications | 233 |
| Economic / value assessment | 53 |

| **Traceability metric** | **Count** |
| --- | --- |
| Core corpus records | 275 |
| Matched to raw export by exact normalized title | 275 |
| Records with a valid DOI | 273 |
| Records with a PMID | 217 |
| Traceable to raw export but without valid DOI or PMID | 1 |
