# Supplementary material for: AI for Health Care Quality and Patient Safety: Scoping Review of Diagnostic, Predictive, and Decision Support Applications
Source: J Med Internet Res. 2026 Sep 21;28:e95157. doi: 10.2196/95157 (PMC13592239; doi:10.2196/95157)
Supplement: Supplementary file 2 [file jmir-v28-e95157-s004.docx]

**Multimedia Appendix 4. Illustrative Primary and Secondary Evidence Across Application Domains**

**This appendix presents 16 illustrative records selected from the 275-record core charting corpus to provide concrete examples of the evidence patterns summarized in the main manuscript. The set was purposively constructed to cover all 4 non-mutually coded application domains and to illustrate complementary evidence functions. Primary empirical and economic records were selected to demonstrate features such as external or multicenter validation, prospective or randomized evaluation, clinical deployment, workflow or process measurement, and economic or value assessment. Secondary evidence syntheses were selected to demonstrate how systematic reviews, scoping reviews, and meta-analyses summarized broader evidence patterns, implementation gaps, and limitations in validation, equity, workflow, or economic evaluation.**

**The number of records was limited to 16 to provide several concrete examples across the 4 application domains while keeping the supplementary table readable. The records were not selected as a statistically representative sample, were not intended to represent all eligible studies, and were not selected, ordered, ranked, or weighted according to formal study quality, sample size, or risk of bias. The complete 275-record charting corpus is provided in Multimedia Appendix 3.**

**The records are presented separately as (A) primary empirical and economic evidence and (B) secondary evidence syntheses. Sample size is reported as participants, encounters, images, sites, or records for primary evidence. For systematic reviews, scoping reviews, and meta-analyses, the number of included studies and the total or pooled sample size are reported where available from the source record; unavailable values are reported as not stated rather than inferred. The four formal economic evaluations identified in the corpus are characterized individually in the Results and are listed in Multimedia Appendix 3.**

**Section A. Primary Empirical and Economic Evidence**

| Domain group | Source | Record ID | Evidence type / design | Sample size or evidence base | AI application / setting | Main charted contribution | Relevant evidence features | DOI / source link |
| --- | --- | --- | --- | --- | --- | --- | --- | --- |
| Diagnostic AI | Castilla et al., 2025 | JMIR-16759 | Retrospective independent external validation | Trained on 275,399 chest X-rays; externally validated on 1045 chest X-rays | AI triage system for chest radiography | Externally validated a commercial CXR triage system for distinguishing normal from abnormal studies and prioritizing urgent cases | External validation; diagnostic workflow relevance | <https://doi.org/10.3390/diagnostics15222899> |
| Diagnostic AI | Hjort et al., 2026 | JMIR-13925 | Development and external validation | 1,142 patients; final external validation set of 71 video recorded procedures involving 104 lesions | AI-supported cystoscopy for bladder cancer detection | Developed and externally validated a real-time AI decision-support tool for bladder lesion detection during cystoscopy | External validation; real-time diagnostic decision support | <https://doi.org/10.1016/j.ijmedinf.2026.106390> |
| Predictive analytics / early warning | Hinson et al., 2022 | JMIR-12800 | Multisite implementation with prospective validation | Retrospective derivation cohort of 21,452 ED patients; prospective validation in 15,670 ED visits across 5 ED sites | EHR-embedded ML system for COVID-19 hospital admission decisions | Implemented a workflow-integrated ML clinical decision support system for short-term deterioration and admission risk | Multisite implementation; prospective validation; workflow integration | <https://doi.org/10.1038/s41746-022-00646-1> |
| Predictive analytics / early warning | Ge et al., 2025 | JMIR-14830 | Development, multiregional validation, and clinical deployment | Three ICU databases: MIMIC-IV n=12,842; eICU n=15,767; Hainan ICU n=210 | ML prediction of moderate-to-severe sepsis-associated acute kidney injury | Developed and externally validated dynamic SA-AKI prediction models across multiregional cohorts and deployed an interpretable tool | Multicenter external validation; clinical deployment | <https://doi.org/10.2196/73840> |
| Predictive analytics / early warning | Park et al., 2026 | JMIR-17873 | Prospective nonrandomized interventional trial | 35,627 general ward admissions; 2906 AI-SaMD alerts | AI-based cardiac arrest prediction model in general wards | Evaluated AI alert-guided reassessment/treatment for ward-based cardiac arrest and in-hospital mortality | Prospective interventional evaluation; alert-mediated workflow | <https://doi.org/10.3390/diagnostics16020335> |
| Clinical decision support / implementation | Shakarbaev, 2026 | JMIR-15341 | Prospective randomized controlled trial | 2384 randomized inpatients across 4 tertiary-care teaching hospitals | AI-enhanced EHR-integrated CDSS for medication safety | Evaluated effects on medication errors, adverse drug events, alert acceptance, clinician adoption, and economic return | RCT; workflow/process metrics; economic indicator | <https://doi.org/10.1016/j.ijmedinf.2026.106457> |
| Economic / value assessment | Ke et al., 2025 | JMIR-17944 | Randomized crossover trial with economic modeling | 14 resident physicians; 272 patient encounters | LLM-based perioperative clinical decision support | Evaluated documentation efficiency, quality, user acceptance, and projected institutional cost savings | Randomized crossover design; economic/value assessment | <https://doi.org/10.1038/s41746-025-01858-x> |

**Section B. Secondary Evidence Syntheses**

| Domain group | Source | Record ID | Evidence type / design | Sample size or evidence base | AI application / setting | Main charted contribution | Relevant evidence features | DOI / source link |
| --- | --- | --- | --- | --- | --- | --- | --- | --- |
| Diagnostic AI | Pervez et al., 2026 | JMIR-18052 | Systematic review and meta-analysis | 17 studies; 11 studies in meta-analysis; >10,000 radiographs | CNNs for pediatric fracture detection on plain radiographs | Synthesized diagnostic accuracy evidence for AI fracture detection in pediatric radiography | Diagnostic meta-analysis; external validation gap identified | <https://doi.org/10.1007/s00330-026-12462-2> |
| Diagnostic AI | Wang et al., 2026 | JMIR-19121 | PRISMA-DTA systematic review and meta-analysis | 6 studies; 4897 observations; 4 studies in primary DTA dataset | AI for burn depth assessment across imaging modalities | Summarized diagnostic accuracy and deployment-readiness features, including validation design and subgroup reporting | Diagnostic accuracy synthesis; deployment-readiness assessment | <https://doi.org/10.1016/j.ijmedinf.2026.106438> |
| Predictive analytics / early warning | Thaitirarot et al., 2026 | JMIR-18401 | Systematic review | 18 studies; 45 prediction models | Machine learning and statistical risk prediction models for new heart failure diagnosis in community settings | Synthesized incident heart-failure prediction models and highlighted external validation and bias limitations | Prediction-model evidence synthesis; external validation gap | <https://doi.org/10.1136/bmjopen-2024-093419> |
| Clinical decision support / implementation | Graafsma et al., 2024 | JMIR-08052 | Scoping review | 10 included studies | AI optimization of medication alerts generated by CDSSs | Mapped AI methods for medication-alert optimization, alert burden reduction, prediction of user responses, and implementation gaps | Workflow/process indicator; alert burden; implementation gap | <https://doi.org/10.1093/jamia/ocae076> |
| Clinical decision support / implementation | Patel et al., 2026 | JMIR-18577 | Systematic review and meta-analysis | 22 studies; >2.3 million patients; 34 sites | Externally validated Epic clinical decision support tools | Synthesized real-world performance of proprietary EHR-integrated CDS tools and compared performance with vendor-reported metrics | External validation synthesis; EHR/CDS implementation | <https://doi.org/10.1007/s11606-026-10381-y> |
| Clinical decision support / implementation | Toh et al., 2024 | JMIR-27438 | Scoping review | 31 studies | CDSSs for spinal disorders | Mapped CDSS functions for imaging avoidance, diagnosis, prognosis, and treatment recommendation in spinal disorders | CDSS evidence synthesis; implementation and external validation needs | <https://doi.org/10.2196/53951> |
| Clinical decision support / implementation | Du et al., 2023 | JMIR-09328 | Systematic review | 17 research papers | ML-based CDSSs for pregnancy care | Identified gaps in explainability, external validation, equity-related reporting, user testing, and implementation | CDSS synthesis; equity/generalizability and implementation gaps | <https://doi.org/10.1016/j.ijmedinf.2023.105040> |
| Economic / value assessment | Lawrence et al., 2025 | JMIR-18783 | Rapid systematic scoping review | 140 included studies; 6 evaluated cost outcomes | AI-supported diagnostics in radiology practice | Synthesized implementation, stakeholder experiences and perceptions, effectiveness, workflow, and cost outcomes for AI-supported radiology diagnostics and identified limited real-world and cost-effectiveness evidence | AI-specific implementation and value synthesis; real-world and cost-effectiveness evidence gaps | <https://doi.org/10.1016/j.eclinm.2025.103228> |
| Economic / value assessment | Alqahtani et al., 2025 | JMIR-16812 | Systematic review | 30 reviewed articles | AI applications in clinical pharmacy | Synthesized AI applications for adverse drug event detection, prescription verification, drug-response prediction, personalized therapy, and cost-effectiveness | Pharmacy safety/value synthesis; prospective validation and cost-effectiveness gap | <https://doi.org/10.1177/20552076251388145> |
