# Supplementary material for: AI for Health Care Quality and Patient Safety: Scoping Review of Diagnostic, Predictive, and Decision Support Applications
Source: J Med Internet Res. 2026 Sep 21;28:e95157. doi: 10.2196/95157 (PMC13592239; doi:10.2196/95157)
Supplement: Supplementary file 3 [file jmir-v28-e95157-s005.doc]

**PRISMA-ScR Checklist**

*Artificial Intelligence for Healthcare Quality and Patient Safety: A Scoping Review of Diagnostic, Predictive, and Decision Support Applications*

| **Section** | **Item** | **PRISMA-ScR Checklist Item** | **Location in Manuscript** |
| --- | --- | --- | --- |
| **TITLE** | | | |
| Title | 1 | Identify the report as a scoping review. | Title page, p. 1. The full title explicitly states "A Scoping Review". |
| **ABSTRACT** | | | |
| Structured summary | 2 | Provide a structured summary that includes (as applicable) background, objectives, eligibility criteria, sources of evidence, charting methods, results, and conclusions. | Abstract, p 1-2, structured with Background, Objective, Methods, Results, and Conclusions sections. |
| **INTRODUCTION** | | | |
| Rationale | 3 | Describe the rationale for the review in the context of what is already known. Explain why the review questions/objectives lend themselves to a scoping review approach. | Introduction p. 2-3, which describes the translational gap in healthcare AI and the need for a cross-domain synthesis, justifying a scoping review approach. |
| Objectives | 4 | Provide an explicit statement of the questions and objectives being addressed with reference to their key elements (e.g., population or participants, concepts, and context). | Review Question under Introduction, p. 3. Explicit questions map AI evidence across diagnostic, predictive, decision-support, and economic or value-assessment domains and identify cross-domain barriers. |
| **METHODS** | | | |
| Protocol and registration | 5 | Indicate whether a review protocol exists; state if and where it can be accessed; and if available, provide registration information including the registration number. | Methods, Protocol, Registration, and Reporting, p. 3. Conducted following JBI methodology and reported per PRISMA-ScR; no protocol was registered. |
| Eligibility criteria | 6 | Specify characteristics of the sources of evidence used as eligibility criteria (e.g., years considered, language, and publication status), and provide a rationale. | Methods, Eligibility Criteria, P. 3-5; Table 1, p. 4-5. Criteria are specified using the Population, Concept, Context framework, including the 2017–2026 time frame, English language, and publication status. |
| Information sources | 7 | Describe all information sources in the search (e.g., databases with dates of coverage and contact with authors to identify additional sources), as well as the date the most recent search was executed. | Methods, Information Sources and Search Strategy, p. 5-6. Five databases (PubMed/MEDLINE, Scopus, Web of Science Core Collection, IEEE Xplore, CINAHL Plus with Full Text); searches were executed on May 18, 2026 and covered records published from January 1, 2017, to April 30, 2026. |
| Search | 8 | Present the full electronic search strategy for at least 1 database, including any limits used, such that it could be repeated. | Methods, Information Sources and Search Strategy, p. 5–6; Multimedia Appendix 2. Full search strategies for all five databases are provided in Multimedia Appendix 2. |
| Selection of sources of evidence | 9 | State the process for selecting sources of evidence (i.e., screening and eligibility) included in the scoping review. | Methods, Selection of Sources of Evidence, p. 6-7; Results, Study Selection and Overview, p. 8-9; Figure 1 and caption, p. 9-10. Staged deterministic rule-based filtering, Stage A scope-signal prioritization, manual title and abstract screening, Stage B evidence-feature prioritization, the final title and abstract based charting prioritization, report-level assessment, and the resulting core charting corpus are described. |
| Data charting process | 10 | Describe the methods of charting data from the included sources of evidence (e.g., calibrated forms or forms that have been tested by the team) and any processes for obtaining and confirming data from investigators. | Methods, Data Charting Process and Data Items, p. 7–8; Multimedia Appendix 3. Structured charting fields, primary charting by one reviewer with subset verification, row-level fields, and count-check procedures are reported. |
| Data items | 11 | List and define all variables for which data were sought and any assumptions and simplifications made. | Methods, Data Charting Process and Data Items, p. 7–8; Multimedia Appendix 3. Variables include application domain, study design or evidence type, validation maturity, deployment or real-world context, workflow metrics, equity or subgroup indicators, infrastructure indicators, governance or lifecycle indicators, and economic or value-assessment characteristics. |
| Critical appraisal of individual sources of evidence | 12 | If done, provide a rationale for conducting a critical appraisal of included sources of evidence; describe the methods used and how this information was used in any data synthesis. | Methods, Methodological Characterization, p. 8. No formal risk-of-bias appraisal or critical-appraisal scoring was conducted; validation maturity and reporting features were charted descriptively, with TRIPOD+AI and PROBAST used only as descriptive lenses. |
| Synthesis of results | 13 | Describe the methods of handling and summarizing the data that were charted. | Methods, Data Charting Process and Data Items, p 7-8; Methods, Synthesis of Results, p. 8; Multimedia Appendix 3. WHO Quality of Care dimensions were used as a deductive scaffold, cross-domain barriers were developed through inductive synthesis, and figure counts were derived from row-level charting fields and count-check procedures. |
| **RESULTS** | | | |
| Selection of sources of evidence | 14 | Give numbers of sources of evidence screened, assessed for eligibility, and included in the review, with reasons for exclusions at each stage, ideally using a flow diagram. | Results, Study Selection and Overview, p. 8-9; Figure 1, p. 9-10. In total, 43,394 records identified, 27,665 records screened, 27,389 excluded or not advanced before report-level assessment, 276 reports assessed at report level, 1 duplicate report removed at final reconciliation, and 275 reports retained in the core charting corpus. |
| Characteristics of sources of evidence | 15 | For each source of evidence, present characteristics for which data were charted and provide the citations. | Results, Study Characteristics and Evidence Landscape, p. 10-11; Table 2, p. 9; Figure 2, p. 10; Multimedia Appendix 3. Characteristics of the 275-record core charting corpus are summarized in the text and visualizations, with the complete row-level charting matrix provided in Multimedia Appendix 3. |
| Critical appraisal within sources of evidence | 16 | If done, present data on critical appraisal of included sources of evidence (see item 12). | Not applicable; no formal risk-of-bias appraisal or critical-appraisal scoring was conducted, consistent with scoping review methodology. Validation maturity was charted descriptively in Methods, Methodological Characterization, p. 7 and Results, Study Characteristics and Evidence Landscape, p. 9-10. |
| Results of individual sources of evidence | 17 | For each included source of evidence, present the relevant data that were charted that relate to the review questions and objectives. | Results, Application-Domain Evidence Patterns, p. 12; Table 2, p. 10; Multimedia Appendix 4; Multimedia Appendix 3. Illustrative records are summarized in Multimedia Appendix 4, and the complete row-level charting matrix for the 275-record core corpus is provided in Multimedia Appendix 3. |
| Synthesis of results | 18 | Summarize and/or present the charting results as they relate to the review questions and objectives. | Results, Cross-Domain Translation Barriers, p. 13-16; Table 3, p. 13; Figure 3, p. 15; Discussion, Principal Findings; Figure 4, p. 16. Charting results are summarized across application domains and synthesized into five cross-domain barriers, with Table 3 defining the barriers, Figure 3 showing indicator visibility, and Figure 4 presenting the conceptual synthesis. |
| **DISCUSSION** | | | |
| Summary of evidence | 19 | Summarize the main results (including an overview of concepts, themes, and types of evidence available), link to the review questions and objectives, and consider the relevance to key groups. | Discussion, Principal Findings and Interpreting the Five Barriers, p 17-19. Summarizes evidence across domains and links to objectives. |
| Limitations | 20 | Discuss the limitations of the scoping review process. | Discussion, Strengths and Limitations, p 19-20. Limitations include lack of prospective registration; no formal medical librarian or information-specialist consultation; single-primary-reviewer screening and charting with subset verification and no fully independent duplicate screening or duplicate data extraction; prioritization-based screening under which some potentially relevant records were retained in the audit trail without being advanced; rapid charting based partly on titles, abstracts, and metadata with targeted manual verification; English-language restriction; absence of supplementary search methods; and no formal risk-of-bias appraisal or meta-analysis, consistent with scoping review methodology. |
| Conclusions | 21 | Provide a general interpretation of the results with respect to the review questions and objectives, as well as potential implications and/or next steps. | Discussion, Conclusions, p 21-22. Five structural barriers; the constraint on clinical value is structural rather than technical; prerequisites for responsible adoption. |
| **FUNDING** | | | |
| Funding | 22 | Describe sources of funding for the included sources of evidence, as well as sources of funding for the scoping review. Describe the role of the funders of the scoping review. | Funding section, p 23. The scoping review was supported by Canada's IDRC (Grant No. 109981-001), IDRC and FCDO (Grant No. 110554-001), and additional named grants, and the funders' roles are described. Funding sources of the individual included studies were not charted. |
